# Recent advances in mitochondria‐targeting theranostic agents

**DOI:** 10.1002/EXP.20230063

**Published:** 2024-03-05

**Authors:** Kun Qian, Shu Gao, Zhaoning Jiang, Qihang Ding, Zhen Cheng

**Affiliations:** ^1^ State Key Laboratory of Drug Research Molecular Imaging Center Shanghai Institute of Materia Medica Chinese Academy of Sciences Shanghai China; ^2^ School of Pharmacy University of Chinese Academy of Sciences Beijing China; ^3^ Shandong Laboratory of Yantai Drug Discovery Bohai Rim Advanced Research Institute for Drug Discovery Yantai Shandong China; ^4^ Department of Chemistry Korea University Seoul Republic of Korea

**Keywords:** disease treatment, medical imaging, mitochondria‐targeting, theranostic agents

## Abstract

For its vital role in maintaining cellular activity and survival, mitochondrion is highly involved in various diseases, and several strategies to target mitochondria have been developed for specific imaging and treatment. Among these approaches, theranostic may realize both diagnosis and therapy with one integrated material, benefiting the simplification of treatment process and candidate drug evaluation. A variety of mitochondria‐targeting theranostic agents have been designed based on the differential structure and composition of mitochondria, which enable more precise localization within cellular mitochondria at disease sites, facilitating the unveiling of pathological information while concurrently performing therapeutic interventions. Here, progress of mitochondria‐targeting theranostic materials reported in recent years along with background information on mitochondria‐targeting and therapy have been briefly summarized, determining to deliver updated status and design ideas in this field to readers.

## Graphical abstract

Possessing diagnostic imaging and therapeutic functions, mitochondria‐targeted theranostic agents can significantly optimize the treatment process and promote precision medicine. This review summarizes the research progress on these agents and the commonly used diagnostic modalities and therapeutic strategies. It also discusses various therapeutic agent categories and their disease applications, addressing unresolved issues and envisioning future directions in the field.

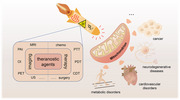

## 1 INTRODUCTION

Mitochondrion, as the power house of cells, is one of the most important organelles that supports the cell's survival and prosperity. Its dysfunction or malfunction is related to a wide spectrum of diseases, including cancer, inflammation, neuron‐degenerative diseases, cardiac disorders, and others,^[^ ^1^ ^]^ which makes mitochondria an important target for diagnosis and treatment. However, mitochondria exist in almost all cells and the number contained in each cell ranges from hundreds to thousands. How to target mitochondria specifically and selectively in lesion site is one of the major difficulties required considerations in the design of mitochondria‐targeting agents.

**FIGURE 1:**
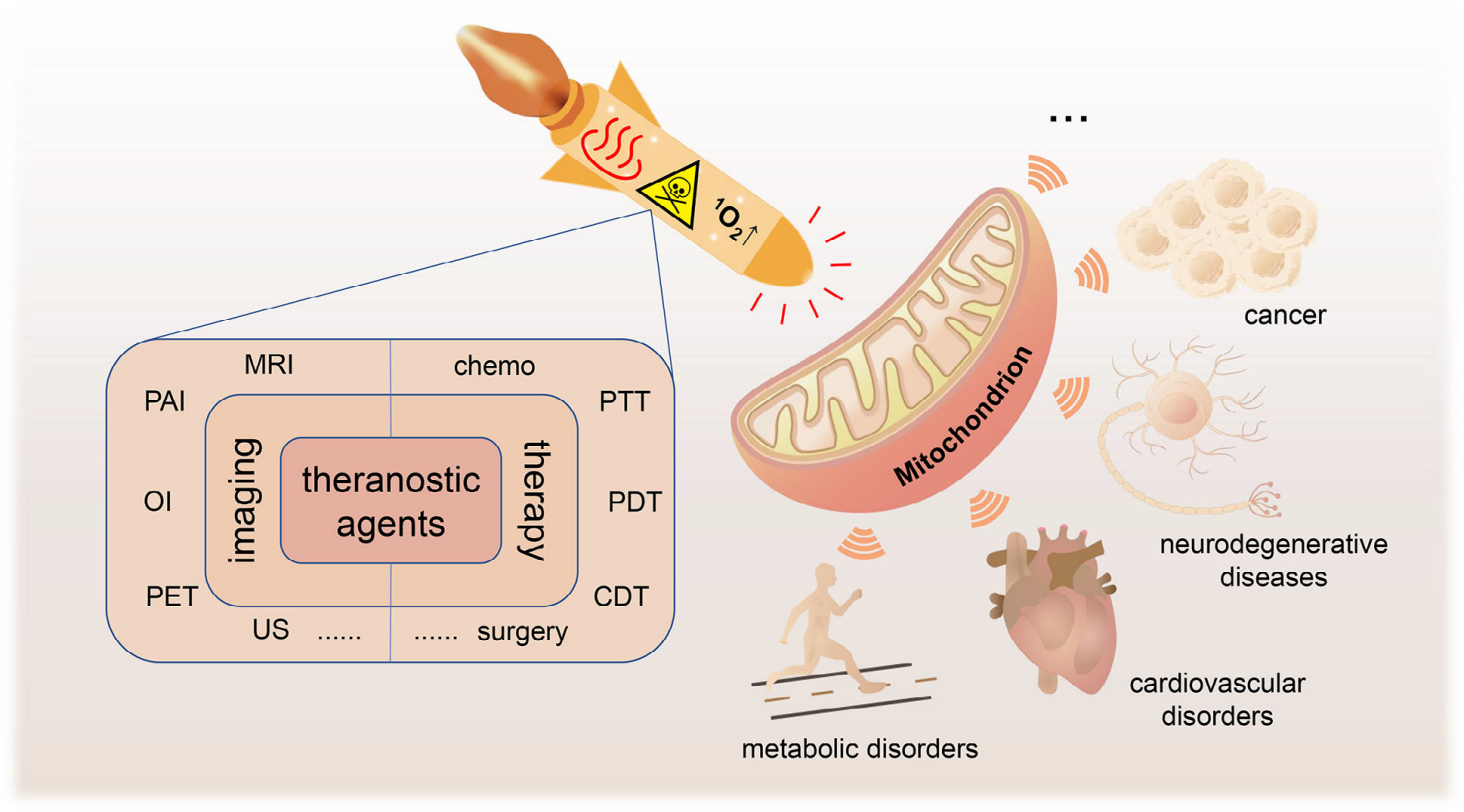
Schematic illustration of mitochondria‐targeting theranostic agents. CDT, chemodynamic therapy; MRI, magnetic resonance imaging; OI, optical imaging; PAI, photoacoustic imaging; PDT, photodynamic therapy; PET, positron emission tomography; PTT, photothermal therapy; US, ultrasound.

Abnormally high mitochondrial membrane potential is one of the characteristics of cancerous mitochondria and have been utilized as driving force for tumor targeting.^[^ ^2^ ^]^ Heart is mitochondria‐rich organ and dysfunction of mitochondria is related to several cardiac diseases.^[^ ^3^ ^]^ Various mitochondria specific probes for optical imaging (OI), positron emission tomography (PET), single photon emission computed tomography (SPECT), magnetic resonance imaging (MRI), etc., have been developed accordingly and successfully applied in imaging of such diseases with good accumulation in interested tissues.^[^ ^4^ ^]^ Mitochondria are not only involved in cellular energy process, but also vital in apoptosis signal pathways.^[^ ^5^ ^]^ The rupture of mitochondria would lead to the release of cytochrome *c* and other factors that trigger the apoptosis process, which has been a strategy for abnormal cell elimination in drug development. Mitochondria‐targeting has also been reported for imaging and treatment of inflammation.^[^ ^6^ ^]^ Therefore, mitochondria would be an ideal target for theranostic with high potential (Figure 1).

Signaling and therapeutic modules are integrated to form theranostic agents that could realize the visualization and treatment of diseases, as well as the monitoring of real time distribution of administered agents and therapy progress. Theranostic is a promising direction for future development of disease treatment, which would optimize and streamline the process of disease treatment and drug discovery by intrinsic drug distribution and therapy outcome monitoring ability.^[^ ^7^ ^]^ In this review, we will mainly focus on the development of mitochondria‐targeting theranostic agents in recent years and their applications in disease diagnosis and treatment. Introduction of mitochondria, related diseases and treatment approaches will be supplemented and followed by the presentation of mitochondria‐targeting theranostic probes categorized by imaging modalities employed. Finally, the perspective and expectation of mitochondria‐targeting theranostic agents will be discussed briefly.

## 2 MITOCHONDRIA AS THERANOSTIC TARGET

### 2.1 Structure and function of mitochondria

Many pieces of evidence currently indicate that mitochondria originated from ancestral alphaproteobacteria‐like organisms and were engulfed by the ancestors of modern eukaryotic cells, establishing a symbiotic relationship.^[^ ^8^ ^]^ They underwent gradual evolution and transformed into what we now refer to as mitochondria, giving rise to the endosymbiotic theory.^[^ ^8^, ^9^ ^]^ Mitochondria are semi‐autonomous organelles with a double membrane structure. They typically exhibit a rod‐shaped morphology, with a length of approximately 1–2 µm, while their morphology can vary depending on their functional adaptations.^[^ ^10^ ^]^

The mitochondria structure, from outer to inner, is sequentially categorized as the outer mitochondrial membrane (OMM), the intermembrane space, the inner mitochondrial membrane (IMM), and the mitochondrial matrix.^[^ ^10^, ^11^ ^]^ The mitochondrial membrane primarily consists of phospholipids and various proteins.^[^ ^12^ ^]^ The OMM serves to separate the mitochondria from the cytoplasm and contains mitochondrial porins to facilitate the passage of ions and small molecules, such as the voltage‐dependent anion channel and some translocases of the outer mitochondrial membrane complexes.^[^ ^13^ ^]^ The IMM is enriched with nonbilayer‐forming phospholipids and specialized proteins.^[^ ^13d^ ^]^ Selective transport of specific ions and molecules across this membrane is facilitated by specific membrane transport proteins. For instance, the adenosine triphosphate (ATP)/adenosine diphosphate (ADP) carrier assists in the movement of ADP across the inner membrane while exporting ATP from the mitochondrial matrix. The IMM plays a critical role in oxidative phosphorylation (OXPHOS), housing four protein complexes embedded within its structure. These complexes include complex I (nicotinamide adenine dinucleotide (NAD) hydride (NADH):ubiquinone oxidoreductase), complex II (succinate dehydrogenase), dimeric complex III (cytochrome *bc*_1_ oxidoreductase), and complex IV (cytochrome *c* oxidase). Additionally, two mobile electron carriers, ubiquinone and soluble cytochrome *c*, are essential components of the electron transport chain.^[^ ^14^ ^]^ The respiratory chain, together with the Krebs' cycle, operates within the IMM, establishing an electrochemical gradient. This gradient not only drives ATP synthesis by complex V ATP synthase but also plays a crucial role in various other functions, such as calcium ion homeostasis.^[^ ^15^ ^]^

The mitochondrial matrix, enclosed by the IMM, serves as the primary site for metabolic pathways. It contains a variety of functional enzymes and houses mitochondrial DNA (mtDNA), RNA molecules, and mito‐ribosomes responsible for mitochondrial protein synthesis.^[^ ^16^ ^]^ The human mitochondrial genome, spanning 16 kilobases, encodes genetic information for 13 hydrophobic proteins essential for respiratory chain complex assembly, while the majority of other components involved in OXPHOS are encoded by the nuclear genome.^[^ ^15^, ^17^ ^]^ To increase the membrane surface area and accommodate a greater number of reactions, the IMM forms invaginations known as cristae. These unique structural features are used to identify mitochondria and their density correlates with the concentration of cytochromes and other electron transfer components, underscoring their significance in mitochondrial function.^[^ ^13^, ^18^ ^]^

Mitochondrial dynamics, which encompass fusion and fission processes occurring within cells, are crucial for reshaping the mitochondrial network and maintaining overall health and functionality of mitochondria.^[^ ^19^ ^]^ Mitochondrial fission involves the division of mitochondria into two separate entities and is primarily mediated by dynamin‐related protein 1.^[^ ^20^ ^]^ Conversely, mitochondrial fusion promotes the interconnection of mitochondria within the network. This process involves two steps: outer membrane fusion, mediated by mitofusin 1 and 2, and inner membrane fusion, mediated by the dynamin‐related GTPase protein optic atrophy 1. These proteins are members of the dynamin‐related GTPases family and play crucial roles in regulating mitochondrial dynamics.^[^ ^21^ ^]^

### 2.2 Mitochondria related diseases and theranostic targeting strategy

Mitochondria represent a fundamental and paramount organelle by empowering cellular activities. They play a pivotal role in supplying energy for diverse cellular activities and actively participate in the regulation of a plethora of cellular processes, including cellular metabolism, apoptosis, cell differentiation, and signal transduction.

Due to the essential nature of mitochondria, cells allocate significant resources and conditions to ensure their stable function. Proteomics, genomics, and bioinformatics have provided a comprehensive understanding of mitochondrial proteins in various eukaryotic organisms.^[^ ^22^ ^]^ Mammalian mitochondria consist of over 1500 distinct proteins, with variations in protein composition among different species to meet specific organismal requirements.^[^ ^23^ ^]^

During evolution, mitochondria have transferred numerous genes to the cell nucleus, retaining genetic information for only 13 proteins. Consequently, mitochondria depend on the cell nucleus and other cellular organelles for the synthesis of most proteins and lipids. In animals, mitochondrial genes are predominantly inherited through the maternal line, while paternal mitochondria are selectively eliminated during embryonic development.^[^ ^24^ ^]^ Mutations in the same mitochondrial protein complex can result in different phenotypes, exhibiting both clinical heterogeneity and tissue specificity in mitochondrial diseases. Both mutations in mtDNA genes and nuclear genes encoding proteins essential for aerobic ATP production can lead to severe, and sometimes catastrophic, mitochondrial diseases in humans. Mitochondrial disorders encompass a broad range of genetically heterogeneous diseases that span across various medical disciplines. Multiple organs might be affected by these disorders at different ages, and exhibit different inheritance patterns, including autosomal, X‐linked, or maternal transmission.^[^ ^25^ ^]^

Mitochondrial structural abnormalities and dysfunction, including disruption of mitochondrial complexes, mitochondrial uncoupling, cristae remodeling, and mitochondrial swelling, lead to the accumulation of reactive oxygen species (ROS), energy stress, cell death, or uncontrolled proliferation. These dysfunctions adversely affect the normal supply of cellular bioenergetics, resulting in systemic dysregulation of organismal functions, thereby contributing to the development of neurodegenerative diseases, cardiovascular disorders, metabolic disorders, and cancer, among others.^[^ ^16^, ^26^ ^]^

#### 2.2.1 Mitochondria in neurodegenerative, renal, and cardiac diseases

Mitochondrial dysfunction is implicated in many neurodegenerative diseases, including Parkinson's and Alzheimer's disease. For instance, in Alzheimer's disease patients, mitochondrial abnormalities such as reduced diameter and surface area, altered morphology, and defective mitochondrial complexes I, IV (cytochrome *c* oxidase), and V (ATP synthase) have been observed. Rare cases of juvenile‐onset Parkinson's disease are associated with mutations in mitochondrial quality control proteins like PINK1 and Parkin, as well as LRRK2.^[^ ^27^ ^]^

Sirtuin 3 (SIRT3), a nuclear‐encoded NAD+‐dependent deacetylase, is predominantly found in the mitochondrial matrix and plays a crucial role in multiple cellular processes, including the tricarboxylic acid cycle, fatty acid β‐oxidation and antioxidant pathways.^[^ ^28^ ^]^ Excessive oxidative stress and mitochondrial damage can lead to decreased SIRT3 levels in tubular cells rich in mitochondria, contributing to acute kidney injury. Enhancing SIRT3 to improve mitochondrial dynamics holds promise as a therapeutic strategy to enhance renal recovery following kidney injury.^[^ ^29^ ^]^

Ischemia‐reperfusion injury refers to the damage that tissues or organs sustain when blood supply is restored after a period of inadequate perfusion.^[^ ^30^ ^]^ Mitochondria are recognized as pivotal factors in initiating heart ischemia‐reperfusion injury due to their abundance in cardiac myocytes and their role in energy provision. Pathological conditions such as calcium overload, oxidative stress, endoplasmic reticulum stress, and immune responses are triggered or amplified by mitochondrial dysfunction, ultimately leading to apoptosis or necrosis of reperfused cardiac myocytes.^[^ ^31^ ^]^ Mitophagy, the autophagic process of clearing damaged mitochondria, influences cardiac function and myocyte vitality. Phosphorylation of RAB9 at the Ser179 site facilitates the assembly of the ULK‐RAB9‐RIP1‐DRP1 complex, promoting mitochondrial engulfment and protecting the heart against ischemia.^[^ ^32^ ^]^ Parkin regulates necrotic cell death by preventing the opening of mitochondrial permeability transition pores, and cyclophilin‐D has emerged as a novel substrate for Parkin ubiquitination, contributing to cardiac injury protection.^[^ ^33^ ^]^

#### 2.2.2 Mitochondria and cancers

The structure and function of mitochondria in tumor cells often experience significant alterations, and aberrant mitochondria undergo metabolic reprogramming to accommodate the unrestricted proliferation of cancer cells.^[^ ^34^ ^]^ Hence, it is possible to improve the targeting of tumor treatment by designing molecules that can specifically target the structure of mitochondria.^[^ ^35^ ^]^

The mitochondria in tumor cells typically exhibit significant distinctions from normal mitochondria. The mitochondrial membrane potential (Δ*Ψ*_m_ or MMP), generated by the proton pump, plays a crucial role not only in the functionality of ATP synthase but also as the driving force for ion and protein transport, which are essential for healthy mitochondrial function.^[^ ^36^ ^]^ In normal cells, the mitochondrial membrane potential fluctuates between −108 and −159 mV, with a median value of approximately −139 mV, and a range of −130 to −140 mV is considered optimal for ATP production.^[^ ^12^, ^37^ ^]^ Possibly due to the cancer cells' reliance on anaerobic respiration and their limited utilization of MMP, they tend to possess a higher degree of hyperpolarized mitochondrial membrane potential compared to normal cells. The MMP can reach as low as around −220 mV, aiding cancer cells in evading apoptosis and facilitating angiogenesis to cope with hypoxia and tumor expansion.^[^ ^2^ ^]^ Therefore, a variety of targeting molecules with lipophilicity and positive charges, such as delocalized lipophilic cations (DLCs), have been designed to specifically accumulate at mitochondria.^[^ ^38^ ^]^ The lipophilicity would benefit the penetration of bio membranes including mitochondria membrane, while cation form would be attracted to the high Δ*Ψ*_m_ because of electrostatic interaction. As indicated by Nernst equation, more than 10‐fold of these probes could be accumulated in cancerous mitochondria than normal cells.^[^ ^39^ ^]^ Furthermore, certain hydrophilic/biocompatible cationic compounds, such as cationic curved polycyclic aromatic molecules, can also serve as probes for mitochondrial targeting.^[^ ^40^ ^]^

The development and progression of tumors are closely associated with abnormalities in mitochondrial gene expression. Imbalances in the expression of oncogenes and tumor suppressor genes can alter mitochondrial respiration and subsequently impact cellular metabolism. Hypoxia‐inducible factor‐1 (HIF‐1), extensively studied in relation to mitochondrial dysfunction, is a cancer gene highly expressed in various tumors.^[^ ^41^ ^]^ It contributes to tumor proliferation and metastasis. Metabolically, it drives the transition from OXPHOS to aerobic glycolysis, which becomes the predominant energy‐generating process in tumor cells.^[^ ^42^ ^]^ Some drugs exert their anti‐cancer effects by inhibiting HIF‐1.^[^ ^43^ ^]^

The oncogene Myc family is overexpressed at supraphysiological levels in various human tumors. One of the family members, c‐Myc, regulates mitochondrial biogenesis by inducing the expression of genes associated with mitochondrial structure and function.^[^ ^44^ ^]^ It stimulates mitochondrial biogenesis to provide energy and intermediates for cell division and oxidative metabolism.^[^ ^45^ ^]^ One of its target genes, PRDX3, is essential for maintaining normal mitochondrial function.^[^ ^46^ ^]^ Myc directly regulates the TFAM gene, encoding a critical factor involved in mitochondrial transcription and replication.^[^ ^47^ ^]^

Ras mutations occur in up to 30% of cancer cases.^[^ ^48^ ^]^ Mutated Ras genes lead to abnormal activation of Ras proteins, stimulating downstream effectors, increasing cell proliferation, inhibiting differentiation, reprogramming metabolism, suppressing apoptosis, and promoting tumor development. The synergistic effects of mitochondrial dysfunction and oncogenic Ras facilitate intercellular tumor progression.^[^ ^49^ ^]^

The tumor suppressor gene tumor protein p53 (TP53) plays a role in regulating mitochondrial respiration and controlling cellular metabolism.^[^ ^50^ ^]^ TP53 inhibits the transcription of glucose transporter (GLUT) isoforms 1 and 4, reducing GLUT3 expression through the IκB kinase‐nuclear factor (NF)‐κB pathway.^[^ ^51^ ^]^ Consequently, the loss of TP53 gene function significantly contributes to the manifestation of the Warburg effect in cancer cells.^[^ ^52^ ^]^

Cellular oxidative metabolism gives rise to various ROS including singlet oxygen (^1^O_2_), superoxide radicals (O_2_^•−^), hydrogen peroxide (H_2_O_2_), and different free radicals such as hydroxyl radicals (HO^•^), perhydroxyl radicals (HO_2_^•^), and carbonate radicals (CO_3_^•−^).^[^ ^53^ ^]^ The mitochondrial respiratory chain serves as a primary source of ROS, and the production of ROS also acts as a pathway for mitochondrial signal transduction.^[^ ^54^ ^]^ These generated ROS molecules function as secondary messengers involved in cellular signal transduction processes.^[^ ^55^ ^]^ While low levels of ROS function as signaling molecules that promote transcriptional activation and normal cell proliferation, such as NF‐κB and activation protein‐1, elevated levels of ROS can induce gene mutations and DNA structural changes, leading to DNA damage, tumor development and metastasis.^[^ ^56^ ^]^ Cancer cells residing in hypoxic microenvironments often exhibit increased ROS production, which aids in the activation of oncogenes, inactivation of tumor suppressor genes, and inhibition of DNA repair mechanisms.^[^ ^57^ ^]^ ROS can disrupt the expression levels of tumor suppressor genes such as p53, p21, p16, FoxO, Rb, BRCA1/BRCA2, PTEN, and USP28.^[^ ^58^ ^]^ Additionally, mutations in proto‐oncogenes can lead to mitochondrial dysfunction, enhanced ROS expression, and accelerated tumor formation. When ROS levels surpass the threshold for cell death, mitochondrial impairment occurs, resulting in mitochondrial membrane depolarization, release of cytochrome *c*, and initiation of cell death pathways.^[^ ^59^ ^]^ Consequently, controlling ROS levels and utilizing ROS‐responsive strategies have emerged as potential therapeutic approaches to counteract tumorigenesis, aiming to enhance treatment specificity and efficacy.^[^ ^60^ ^]^

#### 2.2.3 Strategies for mitochondria‐targeting

As previously highlighted, the quantity, structure, and physiological homeostasis of mitochondria serve as indicators of cellular physiology, imparting crucial diagnostic implications for diseases. Given the inherent vulnerability of mitochondria, they often display heightened sensitivity to various stimuli. Leveraging this susceptibility, researchers have been motivated to manipulate mitochondrial function, aiming to induce damage or degradation specifically in aberrant cells, ultimately leading to curative effects in disease management.

Targeting technology allows for the differentiation of target regions or substances from unrelated entities, thereby optimizing therapeutic outcomes and minimizing undesired side effects.^[^ ^61^ ^]^ The unique characteristics of abnormal mitochondria offer promising opportunities for targeted design. Common targeting strategies involve the modification of theranostic agents with DLC fragments as mentioned in preceding part of this review, enabling their specific accumulation within the highly polarized IMM. Representative mitochondrial targeting motifs include triphenylphosphonium (TPP), rhodamine, F16, heptamethine dye IR‐780, dequalinium, Ir(III), guanidine and their derivatives.^[^ ^62^ ^]^ Enhancing mitochondrial targeting capacity can be achieved by directly conjugating the targeting moiety to therapeutic molecules or by incorporating the mitochondrial targeting moiety into nanocarriers.^[^ ^63^ ^]^ Small hydrophobic cationic moieties can be readily and flexibly modified to yield compounds targeting mitochondria. However, the typically poor solubility and potential toxicity (phototoxicity) following modification can pose challenges for drug delivery, biocompatibility, and in vivo circulation.^[^ ^64^ ^]^

Peptide/protein modification of active compounds targeting mitochondria represents a relatively safe alternative strategy. The combination of mitochondrial targeting signal peptides (MTS) and nanodelivery techniques holds promise for delivering drugs or large molecules into the mitochondria, particularly in the context of gene therapy.^[^ ^65^ ^]^ Cheng et al. developed spherical micelles called M‐ChiP, possessing dual targeting capabilities for mitochondria and the plasma membrane, utilizing a bioactive peptide segment (rFxrFxrFxr, where r represents d‐arginine and Fx represents l‐cyclohexylalanine).^[^ ^66^ ^]^ Li et al. integrated the mitochondrial targeting sequence ALD5MTS with the cell‐penetrating peptide R8, creating R8MTS, a mitochondria‐targeting hybrid peptide that enhances the targeting ability of nanosystems.^[^ ^67^ ^]^ However, this strategy may occasionally lead to false positive results by targeting other sites, and at times, it might be cleared too rapidly to exert its effect.^[^ ^68^ ^]^ Positive charge modifications could enhance mitochondria‐targeting effectiveness, but might greatly reduce the hydrophilicity of resulted agents, leading to difficulties in synthesis, administration, metabolism, and so on.^[^ ^69^ ^]^

Others, such as aptamers and transition metal complexes like Ir(III) complexes, also hold promising potential for mitochondrial targeting development.^[^ ^70^ ^]^ Additionally, the specific enzymes, abnormal levels of ROS, internal pH, temperature, or redox status can serve as design factors for developing responsive strategies to improve mitochondrial targeting efficiency.^[^ ^71^ ^]^

## 3 TREATMENT APPROACHES

Treatment is one of the two cores of theranostic, and mitochondria are valuable target for treatment of diseases like cancer as they are sensitive to chemical, thermal, or mechanical interruptions that cause electronic potential lost or rupture of mitochondria membrane and release of cytochrome *c* and other apoptosis factors.^[^ ^72^ ^]^ Mitochondria could also function as a target to localize therapeutic materials at lesion sites for non‐mitochondria‐specific therapy, such as imaging‐guided surgery, chemotherapy, radiotherapy, and others.

This section encompasses widely utilized treatment modalities such as novel approaches including phototherapy and dynamic therapy, as well as surgery, chemotherapy, and radiotherapy. Their advantages and potentials in disease theranostic, particularly in combating malignant tumors, will be discussed. Dealing with challenges encountered in cancer treatment, such as metastasis, multidrug resistance, and treatment‐resistant relapse, proves to be difficult with a single approach. Combination therapy becomes pivotal in overcoming these obstacles, offering insights into enhancing the effectiveness of integrated diagnosis and therapy.

### 3.1 Phototherapy

Phototherapy, including photodynamic therapy (PDT) and photothermal therapy (PTT), could be easily integrated into theranostic platforms because a large part of photothermal agents and photosensitizers are fluorescent or phosphorescent materials, making them natural theranostic probes with OI ability.^[^ ^73^ ^]^ This approach has distinct treatment modalities and mechanisms compared to traditional methods, providing researchers with high‐performance weapons in disease therapy.^[^ ^74^ ^]^ There are two ways of producing ROS: the generation of type I free radicals by electron transfer and the generation of type II ^1^O_2_ by energy transfer.^[^ ^75^ ^]^ In PDT, photosensitizers are predominantly used to transfer light energy to surrounding oxygen molecules, producing ROS such as Type II ^1^O_2_ that induce oxidative stress and cell damage.^[^ ^76^ ^]^ High concentrations of ROS can induce cell apoptosis, which needs to be avoided in normal cells but becomes a new therapeutic approach for tumor treatment. The limited diffusion distance of short‐lived ROS restricts their anti‐tumor efficacy. Additionally, PDT requires the involvement of oxygen, but the hypoxic microenvironment at the tumor site can sometimes limit the level of ROS production, reducing effectiveness.^[^ ^77^ ^]^ On the other hand, PTT involves the conversion of photon energy into heat by photothermal agents, directly ablating tumor cells.^[^ ^78^ ^]^ PTT overcomes the limitations of oxygen but requires photothermal agents with good photothermal conversion efficiency (PCE) to achieve sufficient temperature for tumor tissue ablation. Generally, heating to temperatures above 48°C for several minutes will cause irreversible damage to local tumor tissue, but the high temperature still poses a significant risk to normal tissue.^[^ ^74^, ^79^ ^]^ Mitochondria, lysosomes, and Golgi apparatus are suitable targets, and by combining targeting strategies with nanotechnology, the enrichment of photothermal agents and photosensitizers at the tumor site can be maximized, resulting in stronger efficacy with limited drug doses. Photons can not only be used for therapy but also provide useful diagnostic or imaging information. Near‐infrared (NIR) dyes with higher tissue penetration and lower autofluorescence interference have prospects in the integration of photo‐diagnosis and therapy. Through modification with targeting moieties such as TPP or their intrinsic targeting abilities, NIR dyes can accumulate in organelles such as mitochondria, illuminating the tumor area upon excitation and exerting PDT or PTT effects.^[^ ^74^ ^]^ Non‐invasive and temporally controlled phototherapy promotes precise tumor treatment.^[^ ^80^ ^]^

### 3.2 Surgery

Conventional protocols such as surgery, chemotherapy, and radiotherapy are still mainstream in clinical practice for cancer therapy. For early‐stage cancer, particularly solid tumors, surgical resection remains the most effective treatment method.^[^ ^81^ ^]^ Even for severe malignancies like pancreatic cancer, early detection and resection have shown significant extensions in patient survival.^[^ ^82^ ^]^ Precise tumor resection reduces damage to normal tissues and organs, resulting in improved postoperative outcomes.^[^ ^83^ ^]^ The key for precise resection is accurate tumor margin identification. The inclusion of a mitochondria‐targeting moiety provides imaging probes with selective localization capabilities. This feature facilitates their specific enrichment at the tumor site, offering sharp tumor margins for imaging‐guided resection surgery.^[^ ^84^ ^]^ Advancements in imaging technologies such as computed tomography (CT), MRI, SPECT, and PET have aided accurate cancer screening and localization of lesions. In recent years, rapid progress in real‐time imaging capabilities of OI technologies, coupled with tumor‐targeting fluorescent probes, has facilitated the real time localization, identification, and removal of malignant lesions during surgery process, further enhancing the specificity of tumor detection and enabling their application in surgical navigation.^[^ ^85^ ^]^

However, surgical resection may not yield satisfactory results for certain non‐solid tumors, such as primary cardiac lymphoma, necessitating alternative methods such as chemotherapy and radiation therapy.^[^ ^86^ ^]^ Furthermore, surgical treatment carries the risk of establishing new tumor metastases or accelerating the growth of residual remnants.^[^ ^87^ ^]^

### 3.3 Chemotherapy

Chemotherapy participates in comprehensive cancer therapy at various stages as a stand‐alone treatment or as an adjunctive measure, particularly in cases of metastatic tumors.^[^ ^88^ ^]^ Chemotherapeutic drugs typically act through two mechanisms to achieve anti‐tumor efficacy: either by affecting the DNA of cancer cells, disrupting DNA replication and repair processes to impede cell growth and division, or by influencing the metabolic processes of cancer cells, disrupting their energy supply and survival mechanisms.^[^ ^89^ ^]^ The use of adjuvants, such as low‐dose pharmacological PFKFB3‐blocking agents like 3PO, can promote tumor vascular normalization and reduce metastasis, thereby enhancing the efficacy of chemotherapy.^[^ ^90^ ^]^ After chemotherapy, there is a possibility of recurrence. A case is that colon cancer cells, upon administration of 5‐fluorouracil, can produce high level of CXCL4, a chemokine that impede anti‐tumor immunity and fosters tumor relapse.^[^ ^91^ ^]^ Resistance to chemotherapy often emerges, such as drug efflux pumps represented by P‐glycoprotein, which hinder the entry of drugs into cells to exert their therapeutic effects.^[^ ^92^ ^]^ With the advent of various strategies, such as ROS response, co‐delivery of drugs, and tumor microenvironment (TME) response, the effectiveness of chemotherapy continues to improve, offering hope for overcoming multidrug resistance and achieving more precise and efficient treatment.^[^ ^93^ ^]^

### 3.4 Radiation therapy

Radiation therapy encompasses external beam radiation therapy and internal radiation therapy with radioactive isotopes, aiming to combat cancer by generating free radicals that cause DNA damage in cells or disrupt cell structures, ultimately leading to a substantial number of cell deaths.^[^ ^94^ ^]^ Unlike chemotherapy, radiation therapy enables localized treatment within the tumor area, thereby reducing systemic toxicity.^[^ ^94b^ ^]^ However, ionizing radiation also exerts toxic effects on normal tissues, limiting the radiation dose for treatment and underscoring the importance of radioprotectors and mitigators.^[^ ^95^ ^]^ Long‐term ionizing radiation exposure brings numerous side effects, such as hair loss, diarrhea, bone marrow suppression, and hematuria.^[^ ^96^ ^]^ Additionally, the hypoxic microenvironment of tumors, which reduces oxygen‐dependent DNA damage, along with the activation of certain immunosuppressive pathways, can influence the efficacy of radiation therapy.^[^ ^97^ ^]^

### 3.5 Others—chemodynamic therapy (CDT) and sonodynamic therapy (SDT)

As mentioned earlier, ROS‐based treatment modalities have gained significant attention.^[^ ^98^ ^]^ In addition to PDT, there are also emerging methods called CDT and SDT. CDT triggers cell apoptosis and inhibits tumor growth by initiating a Fenton or Fenton‐like reaction with an excessive amount of H_2_O_2_ within the tumor tissue, generating hydroxyl radicals.^[^ ^99^ ^]^ CDT utilizes endogenous chemical energy to induce ROS burst and cell apoptosis without the need for light irradiation or ultrasound (US).^[^ ^100^ ^]^ Furthermore, CDT demonstrates higher selectivity as the alkaline environment and low levels of H_2_O_2_ in normal tissues inhibit the Fenton reaction, reducing damage to normal tissues.^[^ ^99^ ^]^

US is a common imaging modality that can also play a role in the field of therapy using sonosensitizers.^[^ ^101^ ^]^ SDT catalyzes the production of ROS through sonosensitizers under US stimulation.^[^ ^102^ ^]^ Compared to PDT, SDT has the advantages of deeper penetration and lower invasiveness, making it applicable for non‐invasive treatment of deep‐seated tumors.^[^ ^103^ ^]^ SDT provides a non‐invasive, targeted treatment approach for solid tumors; however, suitable sonosensitizers still need to be developed.^[^ ^104^ ^]^

### 3.6 The relationship between treatment modalities

In multimodal therapy, each therapeutic approach has its own advances, and they can either function independently or complement each other. Mitochondria serve as the primary sites for intracellular oxidative phosphorylation, offering an opportunity for dynamic therapy. PDT, CDT, and SDT employ distinct ROS initiation methods and penetration capabilities.^[^ ^105^ ^]^

Mitochondria, especially those in tumor cells, exhibit exceptional sensitivity to heat, forming the foundation of PTT. Oxygen‐independent PTT can function either independently or synergistically with dynamic therapy to strengthen anti‐tumor effects.^[^ ^106^ ^]^ Some photosensitizers possess both the efficacy of PDT and PTT, and there are also some probes that have intrinsic cytotoxicity, which can exert chemotherapy effects concurrently with phototherapy or other therapeutic approaches with higher tumor elimination abilities.^[^ ^107^ ^]^

Depending on the specific circumstances, these methods can restrict the tumor region, creating conditions suitable for surgery, radiation therapy, or other therapies. Various treatments can synergize with each other to offer precise and effective theranostic solutions.

## 4 MITOCHONDRIA‐TARGETING THERANOSTIC MATERIALS

The integration of diagnostics and therapeutics has given rise to the field of theranostics. Theranostic agents could simultaneously perform imaging, detection of disease biomarkers, drug delivery efficiency, in situ treatment and outcome evaluation.^[^ ^108^ ^]^ The significance lies in optimizing treatment plans and reducing the likelihood of errors, thereby granting patients more cure rate or survival time. Hence, these dual‐functionality approaches have been rapidly advanced in recent years.

**FIGURE 2:**
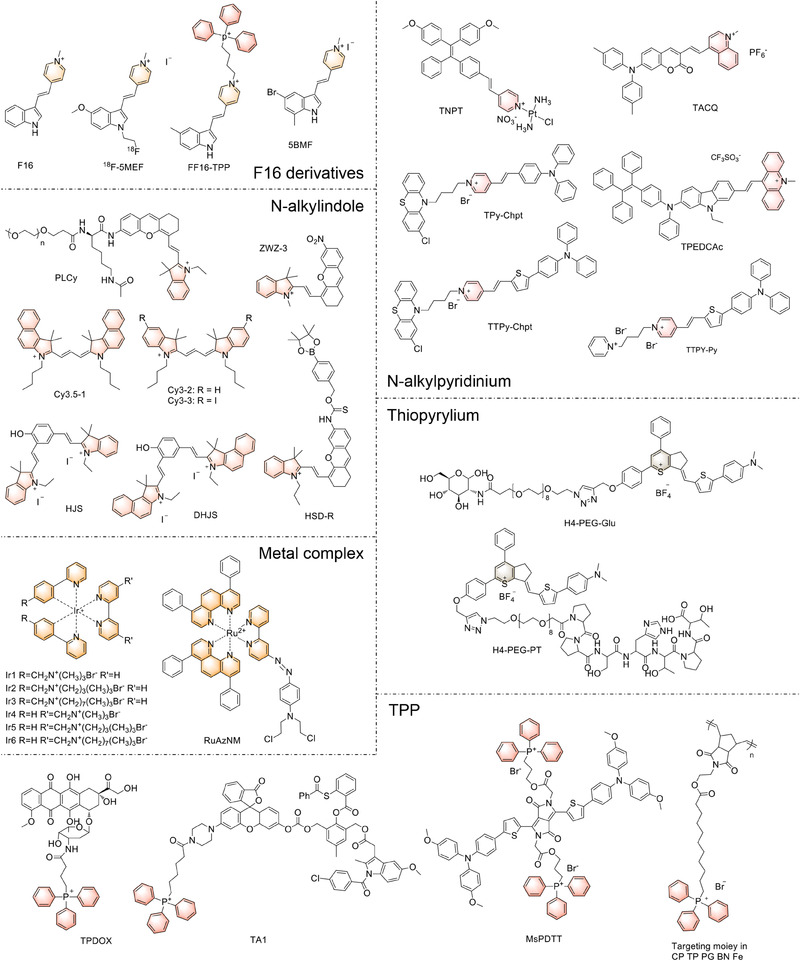
Mitochondria‐targeting molecules categorized by the primary targeting moieties (highlighted in colors). TPP, triphenylphosphonium.

As emphasized earlier, the relationship between mitochondria and various diseases has been highlighted. The development of mitochondria‐targeting theranostic agents holds promise for enhancing the level of treatment for cancers and other diseases, deepening our understanding of the mechanisms involved in mitochondria‐related disorders. In recent years, numerous novel mitochondria‐targeting theranostic agents have emerged. Depending on the imaging modality, they can be categorized into fluorescence probes, photoacoustic (PA) probes, magnetic probes, and multimodal imaging probes. The following sections will provide an overview of recent research progress in mitochondria‐targeting theranostic probes from the perspective of different imaging modalities, and several mitochondria‐targeting organic structures employed in these works have been summarized in Figure 2 and Table 1.

**TABLE 1:** Summarization of representative mitochondria‐targeting theranostic agents.

Agent	Mitochondria‐targeting unit	Disease type/cell line (if reported)	Imaging‐model	Therapy	Remark	Ref.
5BMF	*N*‐alkylpyridinium	Non‐small‐cell lung cancer/H838, H2228, A549 and others	FI	Chemotherapy	Exceptional toxicity	^[^ ^119^ ^]^
5BMF@HSA		Human glioblastoma/U87MG Human breast cancer/MDA‐MB‐231	FI	Chemotherapy	Exceptional toxicity	^[^ ^120^ ^]^
TNPT		Rat glioma/C6	FI	PDT and chemotherapy	All‐in‐one and AIE	^[^ ^121^ ^]^
TPy‐Chpt		Human prostate cancer/PC‐3 Human cervical cancer/HeLa	NIR FI	PDT and dark toxicity	Long‐term near‐infrared imaging	^[^ ^122^ ^]^
TTPy‐Chpt		Human prostate cancer/PC‐3 Human cervical cancer/HeLa	NIR FI	PDT	Long‐term near‐infrared imaging	^[^ ^122^ ^]^
TACQ		Colon cancer/CT26	NIR FI and photothermal imaging	Chemotherapy, PTT, and PDT	Good photothermal conversion efficiencies	^[^ ^73a^ ^]^
PLCy	*N*‐alkylindole	Mouse breast cancer/4T1 Human breast adenocarcinoma/MCF‐7	NIR FI	Dynamic therapy	Sequential enzyme activation	^[^ ^124^ ^]^
ZWZ‐3		Melanoma/B16 and A375	FI	Chemotherapy	Apoptosis and autophagy are induced in a variety of ways	^[^ ^125^ ^]^
Cy3‐3		Human cervical cancer/HeLa	FI	PDT	Mito‐extension	^[^ ^126^ ^]^
HJS		Human prostate cancer/PC‐3 Human cervical cancer/HeLa	NIR FI	Chemotherapy, PTT, and photodynamic combined therapy	Long‐term near‐infrared imaging and AIE	^[^ ^73b^ ^]^
DHJS		Human prostate cancer /PC‐3 Human cervical cancer/HeLa	NIR FI	Chemotherapy, PTT and photodynamic combined therapy	Long‐term near‐infrared imaging and AIE	^[^ ^73b^ ^]^
HSD‐R		Myocardial infarction	FI	Effective cardioprotective efficacies against myocardial ischemia injury	Multiple pharmacological effects including anti‐apoptotic, anti‐inflammatory, and pro‐angiogenic activities	^[^ ^127^ ^]^
H4–PEG‐Glu	Thiopyrylium	Acute myeloid leukemia/THP‐1 and Molm‐13	NIR‐II FI	Chemo‐ and PTT	All‐in‐one and NIR‐II imaging	^[^ ^128^ ^]^
H4‐PEG‐PT		Osteosarcoma/143B	NIR‐II FI	PTT	Water solubility and frequency upconversion luminescence	^[^ ^129^ ^]^
T‐IPIC NPs	TPP	Human cervical cancer/HeLa	NIR‐II FI	PTT & PDT	Only a single‐dose injection	^[^ ^131^ ^]^
TA1		Acute liver injury	FI	Inhibit COX‐2 levels in live cells and PGE2 levels in serum	Two‐photon excited fluorescence imaging	^[^ ^6^ ^]^
PT‐V@TPDOX		Human breast adenocarcinoma/MCF‐7 and its multidrug‐resistant counterpart MCF‐7/ADR	PA imaging	PTT and chemotherapy	Potent therapeutic capability overcoming multidrug resistance	^[^ ^143^ ^]^
CP TP PG BN Fe		Human cervical cancer/HeLa	MRI	Chemotherapy	Receptor‐mitochondria dual‐targeted	^[^ ^148^ ^]^
MsPDTT NPs		Mouse breast cancer/4T1	PAI and NIR FI	PDT and PTT	Hypoxic tumor photoablation	^[^ ^155^ ^]^
AuND‐TPP‐ICG@MCM		Human breast cancer/MDA‐MB‐231	NIR FI, PAI, and SERS imaging	NIR‐II PTT and NIR‐I PDT	Excellent tumor eradication ability	^[^ ^164^ ^]^
Ir3	Gemini Iridium(III) complex	Human hepatocellular carcinoma/HepG2 Human breast adenocarcinoma/MCF‐7	FI	PDT	High water solubility and self‐assembly properties	^[^ ^134^ ^]^
RuAzNM	Ru(II) complex	Human hepatocellular carcinoma/HepG2 and hypoxic HepG2	Phosphorescence imaging	PDT	High selectivity for tumor cells	^[^ ^135^ ^]^
AUC‐GOx/Cel	Unspecified	Mouse breast cancer/4T1	NIR‐II FI	Chemodynamic therapy and starvation therapy	Dual H_2_O_2_ amplification strategy enhances intracellular H_2_O_2_ levels	^[^ ^137^ ^]^
TPEDCAc	*N*‐alkylpyridinium	Human breast adenocarcinoma/MCF‐7	NIR‐II FI, PAI, and Photothermal imaging	PDT and PTT	NIR‐II AIE‐based multi‐modal cancer theranostic system	^[^ ^153^ ^]^
TTPY‐Py⊂CP5@AuNR		Mouse breast cancer/4T1	FI and PAI	PDT and PTT	Temperature and pH‐responsive fluorescence signals	^[^ ^154^ ^]^
mdGC	Unspecified	Mouse breast cancer/4T1	PAI and FI	Mitochondria‐mediated toxicity	Generating ROS without the need for external laser source	^[^ ^157^ ^]^
IR780@Pt NPs	IR780	Osteosarcoma/143B	NIR FI and PAI	Chemophototherapy	Excellent targeting ability	^[^ ^159^ ^]^
IR825@B‐PPNs	IR825	Anaplastic thyroid carcinoma/C643	US, PAI, and FI	PTT combined with antiangiogenesis	Sequential targeting properties	^[^ ^165^ ^]^
ICG@cur‐Gd NPs	Curcumin	Mouse breast cancer/4T1	MRI and FI	Chemo‐/PDT	Tumor‐acidity‐triggered degradation	^[^ ^161^ ^]^
AIPH/ICG@Fe‐PDAP,	Positive charge	Mouse breast cancer/4T1	MRI, PAI, and FI	Free radicals‐based therapy and PTT	Powerful tumor inhibition rate and achieving “CAPIR cascade” in a hierarchical targeting	^[^ ^169^ ^]^
PHPMR NPs	Hematoporphyrin monomethyl ether (assumed)	Atherosclerotic plaque neovascularization	MRI, PAI, and US imaging	SDT	Low‐intensity focused US ‐responsive	^[^ ^170^ ^]^

Abbreviations: FI, Fluorescence Imaging; MRI, Magnetic Resonance Imaging; PAI, Photoacoustic Imaging; PDT, Photodynamic Therapy; PTT, Photothermal Therapy; SDT, Sonodynamic Therapy; SERS, Surface‐Enhanced Raman Scattering; US, Ultrasound.

### 4.1 Theranostic agents for optical imaging

As a rapidly advancing biomedical imaging modality, OI has garnered significant attention in recent years and consists of multiple sub‐categories, including fluorescence imaging, phosphorescence imaging, Raman scattering imaging, etc. The underlying mechanism of fluorescence and phosphorescence both rely on the absorption of excitation photons by imaging probes, and the electron deactivation pathway for the former one is from singlet excited state S_1_ to ground state S_0_ while for the latter is from triplet T_1_ to S_0_. The difference in radiation transition process decided that phosphorescence usually have a much longer lifetime and lower intensity than fluorescence.^[^ ^109^ ^]^ Incident photons are also involved in Raman scattering imaging, but are inelastically scattered rather than absorbed by target molecules or probes. The resulted scattering spectra could provide information on chemical structures of bio samples, making this imaging modality capable of label‐free imaging of interested bio structure or components.^[^ ^110^ ^]^

Diverse optical probes could not only provide insights into relevant tissue structures but also offer valuable information on the functionality of biological entities.^[^ ^111^ ^]^ In contrast to other imaging techniques like CT, PET, and SPECT, OI poses no radiation hazards, exhibits superior biocompatibility and overcomes the limitations associated with tomography, including specificity issues, limited spatiotemporal resolution, low signal‐to‐noise ratio (SNR), high costs, and long acquisition time.^[^ ^111^, ^112^ ^]^ The spectral range of widely reported optical materials has predominantly resided within the ultra‐violet, visible light, and near infrared spectrum (200–1000 nm). However, due to substantial photon scattering, absorption, and autofluorescence within biological tissues, in vivo fluorescence imaging experiences decreased resolution and contrast with increasing tissue depth.^[^ ^113^ ^]^

This issue can be mitigated by extending the wavelength range. Photons within near‐infrared II (NIR‐II, 1000–1700 nm) show significantly decreased scattering and absorption by bio‐tissues, and autofluorescence is reduced to a relatively low level.^[^ ^114^ ^]^ NIR‐II imaging techniques enable enhanced fluorescence imaging with augmented contrast at millimeter depths and micrometer resolutions, presenting a promising future for fluorescence imaging.^[^ ^115^ ^]^ Furthermore, NIR‐II imaging plays an indispensable role in multimodal imaging, significantly enhancing imaging and detection accuracy.^[^ ^116^ ^]^

#### 4.1.1 F16, its derivatives, and analogues

F16 represents a paradigmatic mitochondria‐targeted theranostic small molecule that possesses both mitochondria‐targeting cytotoxicity and fluorescence imaging capability through its lipophilic cationic moiety and N‐methylpyridinium linkage. Simultaneously, F16 exhibits potent cytotoxicity against various cancer cell types, leading to extensive investigations in the field of theranostics.^[^ ^117^ ^]^ Zheng et al. conducted a study in which F16 molecular scaffold was utilized to prepare a radiofluorinated probe known as ^18^F‐5MEF.^[^ ^118^ ^]^ This probe was employed for PET and fluorescence dual‐modal imaging of the myocardium, aiming to facilitate the early diagnosis of heart failure and accurate assessment of myocardial viability in various cardiovascular diseases. This innovative application of F16 expanded its biomedical utility into new domains. Wu et al. have successfully synthesized a series of F16 analogues and F16‐TPP conjugates based on alkyl TPP salts and F16, thereby expanding the repertoire of the F16 molecular platform. In comparison to F16, the F16‐TPP conjugate exhibited diminished cellular uptake and cytotoxicity, potentially attributed to the enhanced positive charge, which impeded its permeability across cell membranes. However, FF16 and FF16‐TPP demonstrated heightened efficacy and comparable or increased mitochondrial accumulation within tumor cell lines relative to F16.^[^ ^62b^ ^]^

**FIGURE 3:**
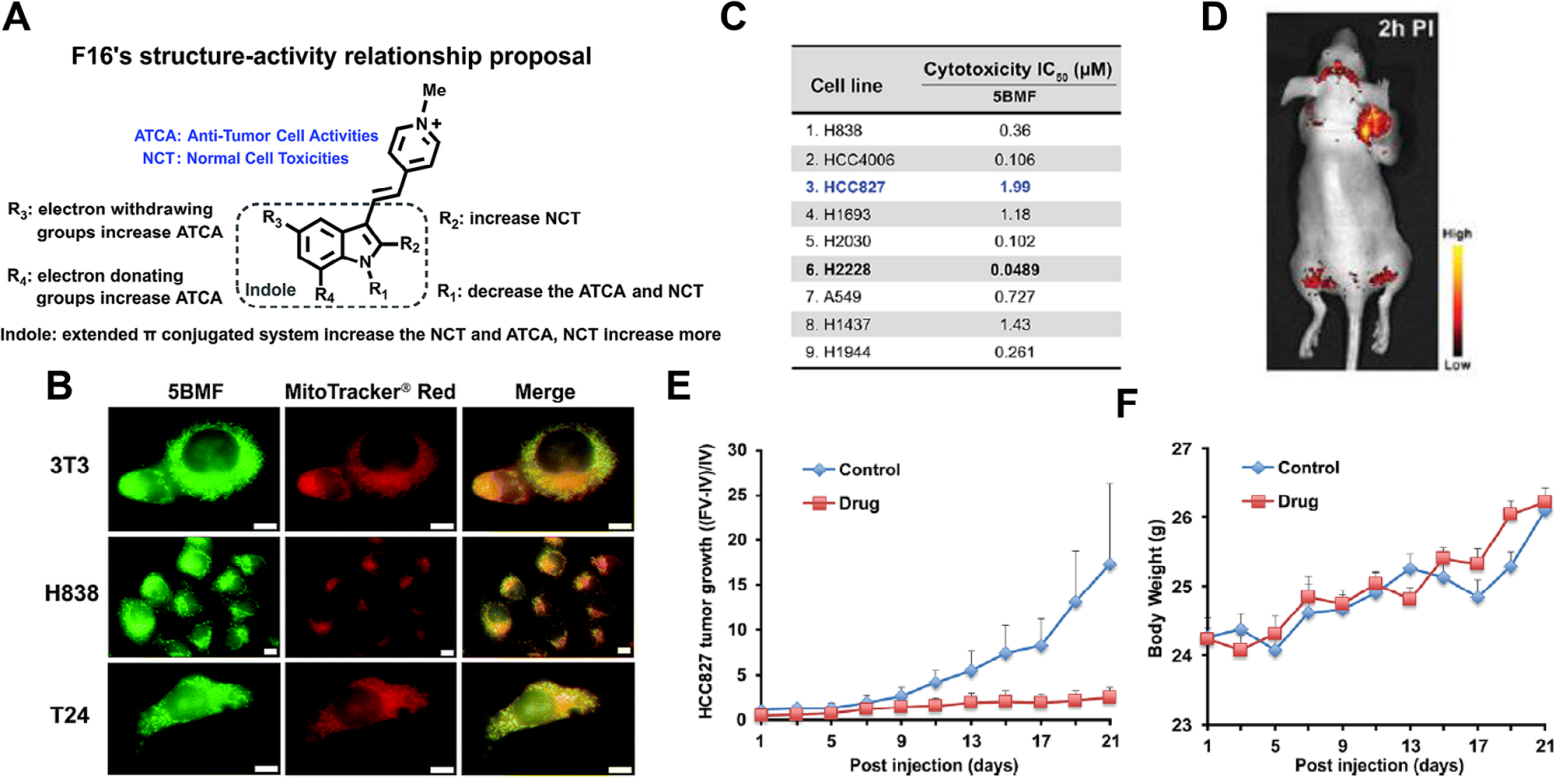
5BMF and its applications in mitochondria‐targeting imaging and tumor treatment. (A) F16s structure‐anti‐tumor activity relationship proposal. (B) Colocalization of 5BMF (green) with mitochondrial specific probe MitoTracker red (red). The Pearson's correlation coefficient (*γ*) for 3T3, H838, and T24 is 0.978, 0.924, and 0.929, respectively. 63× oil objective lens. White scale bar:10 µm. (C) 5BMF's half‐maximal inhibitory concentrations (IC_50_) of 9 lung cancer cell lines. (D) A representative in vivo fluorescence image of a human lung cancer HCC827 bearing mouse 2 h post‐injection of 5BMF (15 mg kg^−1^, 0.306 m kg^−1^; for a 20 g mouse, inject 6.11 mm 5BMF phosphate buffered saline (PBS) solution 150 µL; fluorescence imaging was performed with 450 nm excitation light, and the emission light was collected using a 550 nm long‐pass filter) (*n* = 4). (E) Effects of 5BMF on the tumor growth of HCC827 in nude mice (*n* = 10 per group). FV, final volume. IV, initial volume. (F) Effects of 5BMF on the tumor‐bearing nude mice's body weight (*n* = 10 per group). The treatment was started when the tumors reached ≈3 mm in diameter (day 1). Control group: PBS, Drug group: 5BMF, 15 mg kg^−1^, 0.306 m kg^−1^; for a 20 g mouse, inject 6.11 mm 5BMF PBS solution 150 µL; i.v. injections were given on day 1, 3, 5, 7, 9, 11, 13, 15, 17, 19, and 21. Reproduced under the terms of the Creative Commons Attribution 3.0 Unported License.^[^ ^119^ ^]^ Copyright 2019, the Royal Society of Chemistry.

Chen et al. conducted a study on the structure‐activity relationship of F16 and identified its derivative, 5BMF, as an exceptionally efficient theranostic agent against cancer (Figure 3).^[^ ^119^ ^]^ In vitro cellular experiments demonstrated that 5BMF exhibited superior mitochondria‐targeting ability (Figure 3B), remarkable anticancer activity, and relatively higher selectivity compared to other derivatives. The half‐maximal inhibitory concentrations (IC_50_) of 5BMF against T24 cells and H838 cells were determined to be 0.82 and 0.36 µm, respectively, while F16 exhibited IC_50_ values of 18.8 and 46.6 µm against the same cell lines, respectively. Notably, 5BMF displayed prominent antitumor cell activity in non‐small‐cell‐lung cancer cell lines, encompassing H838, HCC4006, HCC827, H1693, H2030, H2228, A549, H1437, and H1944 (Figure 3C). Specifically, the H2228 cell line exhibited a remarkably low IC_50_ value of 48.9 nm for 5BMF. Moreover, the ratio of the anti‐cancer cell activity to the anti‐normal cell (3T3) activity for 5BMF was approximately 225. In vivo imaging studies substantiated the tumor accumulation ability of 5BMF. Following the intravenous (i.v.) administration of 15 mg kg^−1^ 5BMF in HCC827 tumor‐bearing nude mice, the tumor tissue exhibited high fluorescence intensity, with a tumor‐to‐background ratio of approximately 2, thereby facilitating fluorescence image‐guided surgery (Figure 3D). In a therapeutic regimen on HCC827 tumor‐bearing mice spanning 21 days, the group treated with intermittent i.v. injections of 5BMF displayed a 2‐fold increase in tumor volume, whereas the control group (phosphate buffered saline (PBS)‐treated group) exhibited a 16‐fold increase, underscoring the exceptional anticancer efficacy of 5BMF (Figure 3E,F). The integration of mitochondria‐targeting, fluorescence imaging, and potent and broad‐spectrum anticancer activity within a single small molecule exemplified the F16 family as a promising platform for the development of advanced targeted theranostic.

In addition, a complex of human serum albumin (HSA) and 5BMF, denoted as 5BMF@HSA, has been developed by Qian et al.^[^ ^120^ ^]^ This complex exhibited a remarkable enhancement in the aqueous solubility of 5BMF, achieving an approximately 3.4‐fold increase (from 1.61 to 5.41 mg mL^−1^), accompanied by a more than twofold augmentation in fluorescence intensity. Importantly, the 5BMF@HSA complex retained the outstanding anti‐tumor efficacy of 5BMF and exhibited a higher release of 5BMF under acidic conditions compared to neutral environment. Thus, the 5BMF@HSA complex represented a highly promising and potent candidate for cancer theranostic, demonstrating great potential in the field of oncology.

#### 4.1.2 Positive charges induced by *N*‐alkylpyridinium

N‐methylpyridinium was the key component of F16 molecules to introduce positive charges and several works have utilized this structure as mitochondria‐targeting moiety. Guo et al. integrated pyridinium cation containing aggregation‐induced emission (AIE) fluorophores with cisplatin to synthesize a mitochondria‐targeting photosensitizer, TNPT, which exhibited an integrated theranostic anti‐cancer performance.^[^ ^121^ ^]^ The authors provided detailed insights into the molecular design principles and emphasized the influence of intramolecular charge transfer (ICT) effect and energy gap *Δ*_EST_ on the efficacy of PDT when incorporating D‐A strength. In vitro experiment validated the mitochondria accumulation and imaging ability of TNPT as well as its efficient and selective cytotoxicity toward tumor cells upon white light irradiation. Zhang et al. also reported two *N*‐alkylpyridinium containing AIE luminogens (AIEgens), TPy‐Chpt and TTPy‐Chpt, for long‐term mitochondrial‐targeting NIR imaging and PDT.^[^ ^122^ ^]^ TTPy‐Chpt was obtained by introducing a thiophene moiety as π‐bridge into TPy‐Chpt, which altered the AIEgen's mitochondrial targeting, cell imaging, and cytotoxicity performance. TTPy‐Chpt demonstrated a higher capacity to generate singlet oxygen. Both AIEgens displayed strong phototoxicity against cancer cells, while TPy‐Chpt exhibited superior NIR imaging and mitochondrial targeting capability compared to TTPy‐Chpt. Cellular imaging verified their mitochondria‐targeting ability and demonstrated a sustained cell retention. Seven days after intratumoral injection of AIEgens in PC‐3 tumor‐bearing mice, the in vivo NIR fluorescence still remained strong, indicating the long‐term imaging capability of both AIEgens.

Li et al. developed series mitochondria‐targeting theranostic agents named TACP, TACQ, and TACA, which exhibited AIE ability and regulatory activity towards mitophagy.^[^ ^73a^ ^]^ These agents consisted of two dimethylamino‐substituted coumarin groups as electron‐donating units and employed various nitrogen‐containing aromatic amines as electron‐withdrawing units. The emission wavelength of TACP was in the near infrared I (NIR‐I) region, while those of TACQ and TACA reached the NIR‐II region. These desired fluorophores exhibited typical AIE characteristics owing to the pronounced push‐pull effects and distorted molecular conformations.

Due to its favorable lipophilic cationic properties, TACQ demonstrated selective targeting towards cancer cell mitochondria. Confocal laser scanning microscopy experiments were conducted to assess the cellular uptake and subcellular localization of AIEgens. Further colocalization studies confirmed that TACQ was efficiently internalized by cells and achieved specific mitochondrial targeting within a span of 30 min. Notably, even without the need for washing steps, TACQ exhibited superior image contrast compared to the commercially available mitochondria probe, Mito‐Tracker Green (MTG). Furthermore, TACQ provided enhanced three‐dimensional imaging of mitochondria, with superior lateral resolution when compared to MTG. In co‐culture scenarios where multiple cancer cells and normal cells (U‐87 MG‐fluc‐GFP, MCF‐7, HPCE, and CHO or U‐87 MG‐fluc‐GFP, HPCEC, HPCE, and CHO) were cultivated together, TACQ selectively stained the cancer cells. Benefiting from its exceptional targeting capabilities and inherent AIE properties, TACQ represented a unique imaging agent specifically designed for visualizing cancer‐associated mitochondrial alterations.

Following co‐incubation of HeLa cells with TACQ, the emergence of vacuoles, including dual‐membrane vesicles harboring compromised organelles, and the presence of cellular fragments were observed, demonstrating the formation of autophagosomes.^[^ ^123^ ^]^ Furthermore, Western blot analysis revealed the conversion of LC3‐I to LC3‐II, a well‐established marker protein for autophagy, thus providing evidence that TACQ possessed the ability to induce mitophagy.

TACQ exhibited a PCE of 55% and possesses excellent ^1^O_2_ generation capability. To further evaluate its in vivo imaging and suitability for cancer therapy, a xenograft model was established by implanting CT26 cells into female BALB/c mice. When the tumor volume reached 1000 mm^3^, TACQ (5 mm, 100 µL) was administered by intratumoral injection. Upon irradiation of the tumor tissue with laser (635 nm, 200 mW cm^−2^), the temperature increased from 34.6 to 54.4°C after 10 min of light exposure. After 21 days of treatment involving simultaneous administration of TACQ and same laser irradiation, almost complete tumor ablation was achieved in tumor‐bearing mice.

#### 4.1.3 Cyanine‐like structures

*N*‐alkylindole and its analogues are the fundamental component of cyanine dyes and because of the cationic property, these dyes also exhibit mitochondria accumulation ability. Ma et al. developed a macrotheranostic probe, PLCy, for selective cancer cell mitochondrial targeting imaging and therapy through sequential enzyme activation.^[^ ^124^ ^]^ The probe consisted of an NIR fluorophore, hemicyanine (CyNH2), conjugated with acetylated lysine residues and poly(ethylene glycol) (PEG). In vitro experiments demonstrated the excellent mitochondrial targeting ability of PLCy, leading to mitochondrial membrane damage and subsequent generation of ROS. Its enzymes responsive activity was validated by cellular toxicity evaluation while its in vivo tumor imaging and suppression ability was demonstrated in tumor bearing animal model experiment.

Liu et al. developed a novel hemicyanine‐based fluorescent probe, ZWZ‐3, and investigated its application in imaging and therapy of melanoma.^[^ ^125^ ^]^ Subcellular localization experiments demonstrated that ZWZ‐3 preferentially accumulated in mitochondria with concentration dependence, and cytotoxicity against B16 and A375 cell lines (IC_50_ values are 0.2 and 0.43 µm, respectively) were verified by cellular experiments. Further investigation into its cellular uptake mechanism revealed that ZWZ‐3 selectively accumulated in tumor cell mitochondria through an organic anion transporting polypeptide‐dependent mechanism, resulting in upregulation of Bax, activation of caspase‐3, caspase‐9, and poly (ADP‐ribose) polymerase, induction of mitochondrial membrane depolarization, and generation of ROS. Consistent with the in vitro results, in vivo experiments demonstrated that ZWZ‐3 suppressed melanoma tumor growth, promoted cell apoptosis, and achieved a tumor growth inhibition rate of 76.3% on the 38th day post‐implantation. ZWZ‐3 showed no significant toxicity in vivo, indicating its potential as a safe and effective theranostic agent.

**FIGURE 4:**
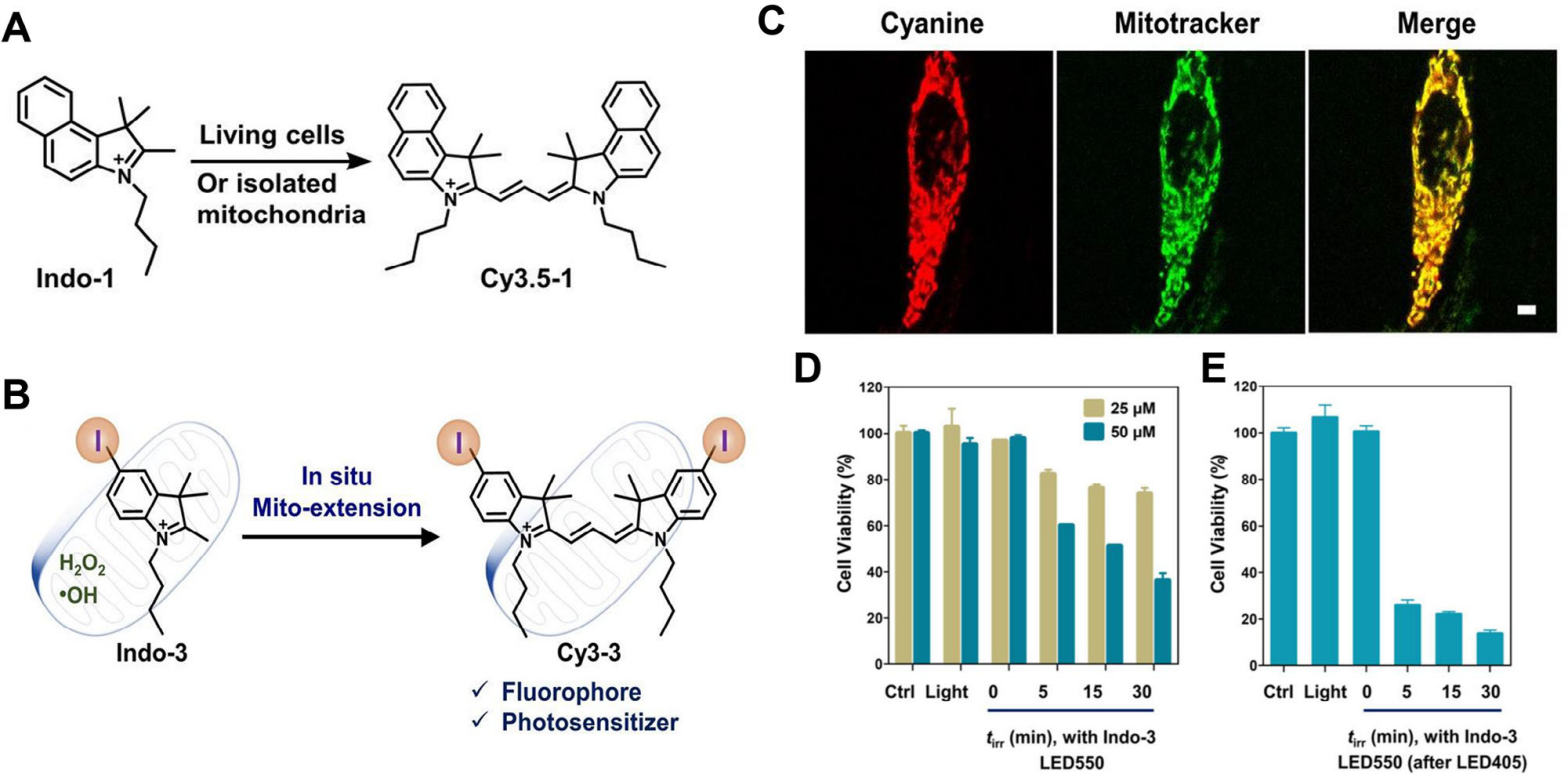
Evaluation of mitochondria responsive theranostic strategy. (A) Schematic presentation of Indo‐1 to Cy3.5‐1 conversion in living cells or isolated mitochondria (^iso^Mito). (B) Schematic presentation of the intracellular effects of Cy3‐3 through mitochondria‐directed extension, fluorescence response and ^1^O_2_ sensitization in the live cells. (C) Confocal laser scanning microscopy images of HeLa cells that were pre‐incubated with Indo‐3 (10 µm) for 12 h in dark and stained with MitoTracker. Scale bar: 5 µm. (D) Viabilities of cells that were pre‐incubated with Indo‐3 (25 µm or 50 µm) for 12 h, light irradiation (550 nm, 25 mW cm^−2^, *t*_irr_ = 5, 15 and 30 min) and incubated for additional 24 h. (E) Viabilities of cells that were pre‐incubated with Indo‐3 (50 µm) for 12 h, light irradiation (405 nm, 35 mW cm^−2^, 30 min; and then 550 nm, 25 mW cm^−2^, *t*_irr_ = 5, 15 and 30 min) and incubated for additional 24 h. Error bars represent the standard deviations of independent MTT measurements (*n* = 3, mean ± SD). Reproduced with permission.^[^ ^126^ ^]^ Copyright 2022, Wiley‐VCH.

Heng et al. devised three synthetic strategies for the production of trimethylated phthalocyanines using 1‐butyl‐2,3,3‐trimethyl‐3H‐indolium (PreCy) derivative as a precursor.^[^ ^126^ ^]^ These strategies include (a) photoextension, (b) inducer‐extension, and (c) Mito‐extension (referred to as mitochondria‐directed extension). Notably, the Mito‐extension approach enabled the utilization of endogenous mitochondrial ROS within live cells for the conversion of cyanine dyes (Figure 4A,B), facilitating cellular imaging and phototherapy while minimizing the reliance on external light or other exogenous assistance.

In the case of Mito‐extension in live cells, the precursors Indo‐1, Indo‐2, and Indo‐3 were co‐incubated with HeLa cells for 1 h, leading to the detection of distinct signals corresponding to Cy3.5‐1, Cy3‐2, and Cy3‐3, respectively. Both Cy3.5‐1 and Cy3‐3 exhibited specific expression within the mitochondria, as evidenced by a Pearson correlation coefficient of 0.86 for co‐localization. Under excitation, Cy3‐3 demonstrated the ability to induce ROS generation. Given predominant accumulation of Indo‐3 in mitochondria (Figure 4C), it suggested the potential of mitochondria to synthesize Cy3‐3 in situ as a targeted photosensitizer for inducing cellular apoptosis (Figure 4D,E). Moreover, noticeable disparities in PreCy conversion levels were observed between normal cells and cancer cells, with elevated ROS levels in tumor cells promoting cyanine expression. This presented a promising opportunity for the application of in situ phthalocyanine synthesis in cancer therapy.

Zhang et al. designed and synthesized two mitochondria‐targeted NIR AIE theranostic agents, HJS and DHJS, possessing both photothermal and photodynamic activities.^[^ ^73b^ ^]^ At low concentrations, they enabled rapid (within 1 min) mitochondria‐targeted red fluorescence imaging with the potential to indicate membrane potential. DHJS demonstrated superior mitochondrial targeting compared to HSJ in SGC‐7901 cells. In vivo experiments demonstrated bright NIR fluorescence observed in PC‐3 tumor‐bearing mice after i.v. administration of DHJS at 0.5 h. The probe showed potential for chemotherapy, PTT, and PDT in cytotoxicity experiments. However, the relatively fast metabolism of DHJS in vivo may have resulted in less pronounced tumor inhibition upon i.v. administration, and direct intratumoral injection of DHJS was necessary to achieve significant tumor suppression. Therefore, it was crucial to enhance the tumor‐targeting capability and in vivo retention of the probe.

*N*‐alkylindole based structure could also be designed as ROS responsive theranostic probes.^[^ ^127^ ^]^ This agent named as HSD‐R was designed for the treatment of myocardial infarction (MI). The HSD‐R, facilitated by its positively charged indole moiety, selectively targeted mitochondria and emitted red fluorescence in the presence of ROS, thereby enabling visualization and quantification of the release kinetics and distribution of hydrogen sulfide. The utilization of a fluorescence moiety with an extended emission wavelength endows HSD‐R with exceptional performance in in vivo imaging and quantification. Following administration to MI rats, HSD‐R transformed into HSD‐RF with fluorescence recovery and release of H_2_S for MI treatment, exhibiting a concentration‐dependent increase in fluorescence intensity that peaked at 2 h post injection. Local injection of HSD‐R exhibited sustained hydrogen sulfide release for over 12 h, effectively counteracting the significant reduction in hydrogen sulfide levels observed in MI rats.

HSD‐R exhibited a spectrum of pharmacological effects, including anti‐apoptotic, anti‐inflammatory, and pro‐angiogenic properties. Its anti‐apoptotic mechanism involved the inhibition of key apoptotic factors such as BID, Apaf‐1, and p53. HSD‐R demonstrated the ability to preserve cardiac contractility, reduce infarct size, improve left ventricular wall thickness, and enhance cardiac function, with more pronounced effects observed at higher doses. Thus, HSD‐R possessed great promise as a theranostic agent for precise treatment of MI and other ischemic diseases.

#### 4.1.4 Cations with thiopyrylium

Benefiting from high spatiotemporal resolution, deeper tissue penetration, and low background autofluorescence, NIR‐II fluorescence imaging technology has been widely employed in various fields such as tumor imaging and image‐guided surgery.^[^ ^114^ ^]^ Zheng et al. synthesized a novel mitochondria‐targeting NIR‐II small molecule probe, H4‐PEG‐Glu, incorporating a sulfur pyridinium cation, for the integrated treatment and imaging of cancer.^[^ ^128^ ^]^

In vitro cell viability experiments demonstrated that H4‐PEG‐Glu exhibited specific cytotoxic effects on acute myeloid leukemia (AML) cells compared to normal cells in the absence of laser irradiation. Moreover, H4‐PEG‐Glu displayed excellent photostability and photothermal effect with a PCE of 11.6%. Under 808 nm laser irradiation, H4‐PEG‐Glu exhibited effective PTT against AML cancer cells (THP‐1 and MOLM‐13) in vitro.

In vivo imaging and tumor treatment experiments were conducted using a patient derived xenograft mouse model derived from AML patients. Results showed the specific targeting ability and good PTT effect of H4‐PEG‐Glu towards AML cells within the bone marrow, suggesting that H4‐PEG‐Glu held promise as a potential small molecule NIR‐II probe for the theranostic application in AML tumor management.

Similar structure has also been reported by this group. Named as H4‐PEG‐PT, this D‐A type thiopyrylium‐based fluorophore exhibited frequency upconversion luminescence (FUCL) at ≈580 nm under ≈850 nm excitation.^[^ ^129^ ^]^ FUCL materials could convert low‐energy excitation into high‐energy emission through anti‐Stokes shift. They possessed properties such as limited autofluorescence from biological samples, high SNR, and controllable excitation and emission, making them applicable in the field of biosensing and imaging.^[^ ^130^ ^]^ In this study, H4‐PEG‐PT could work as a DLC thiopyrylium salt at low concentrations, which allows for efficient targeting of mitochondria. Intravenous injection of H4‐PEG‐PT (200 µg, 200 µL) into 143B tumor‐bearing mice resulted in clear visualization of tumor tissue in the NIR‐II images, with a SNR of 4.57 ± 0.15 at 12 h. The PCE of H4‐PEG‐PT was approximately 18%, while the generation of ROS was negligible. Continuous irradiation of H4‐PEG‐PT‐treated tumor‐bearing mice with 808 nm laser (1.5 W cm^−2^) for 8 min led to a rapid increase in tumor surface temperature from 33.0°C to 67.8°C, while the temperature elevation of normal skin was minimal. H4‐PEG‐PT demonstrated potent PTT efficacy against 143B tumors. The design and synthesis of H4‐PEG‐PT enriched and expanded the application of FUCL‐based mitochondrial‐targeted NIR‐II probes in tumor detection/imaging and image‐guided surgery.

#### 4.1.5 TPP cations as targeting moiety

**FIGURE 5:**
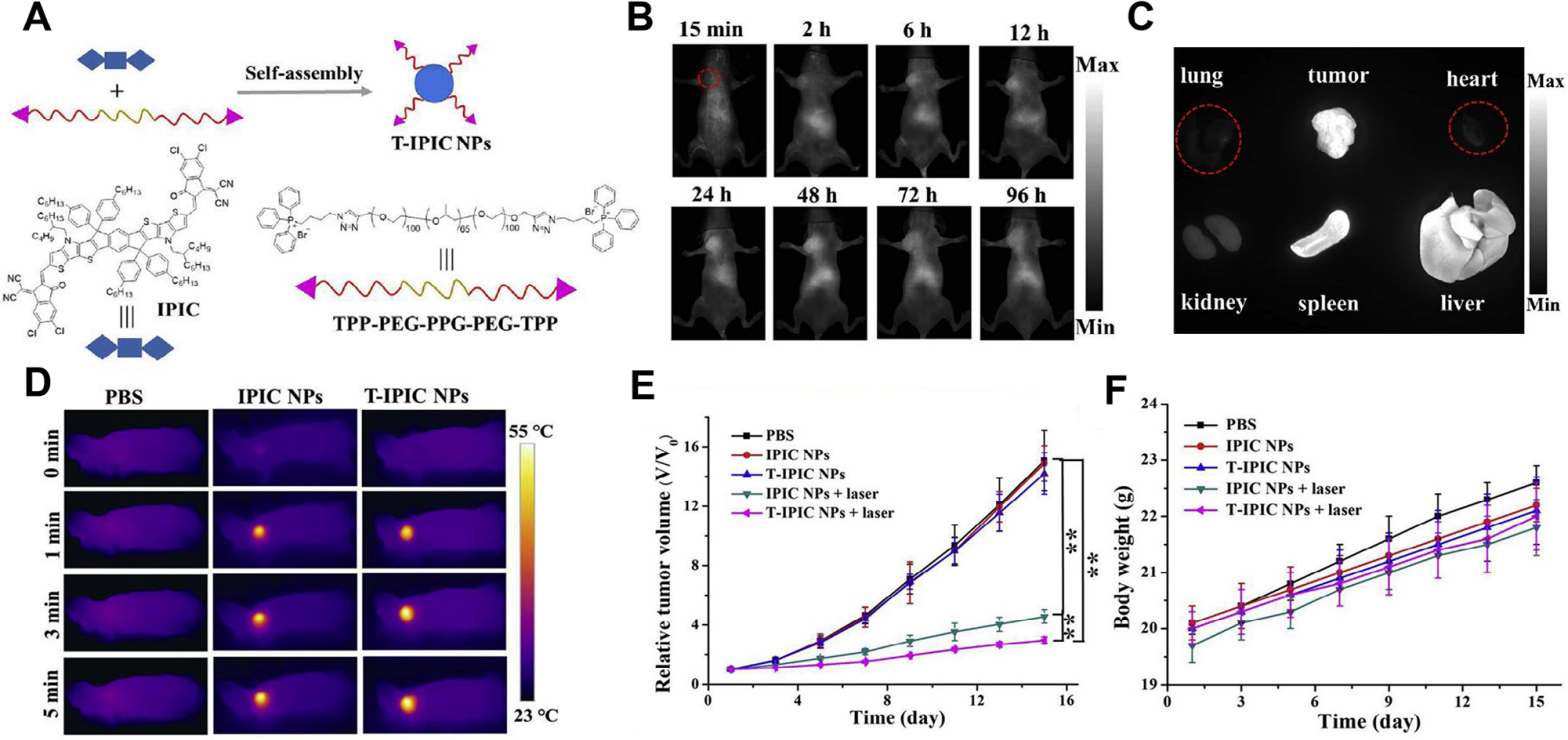
Phototheranostic nanoparticles for cancer treatment. (A) Preparation of T‐IPIC NPs. (B) In vivo near infrared‐II (NIR‐II) fluorescence images of mouse (HeLa tumor model) after injection of T‐IPIC NPs. (C) Ex vivo NIR‐II imaging of tumors and major organs. (D) Photothermal images of mice under 808 nm laser illumination. (E) Relative tumor volumes and (F) body weight curves of mice during treatment (*p < 0.05; **p < 0.01). Reproduced with permission.^[^ ^131^ ^]^ Copyright 2019, Elsevier Ltd.

As a widely accepted and commonly used mitochondria‐targeting moiety, TPP cations are also active in theranostic stage. Based on PEGylated TPPs, Wang et al. developed a novel one‐for‐all phototheranostic nanoparticle system, named T‐IPIC NPs, for precise treatment of cancer through NIR‐II fluorescence imaging‐guided mitochondrial targeting.^[^ ^131^ ^]^ T‐IPIC NPs were formulated using an amphiphilic mitochondria‐targeting TPP‐PEG‐PPG‐PEG‐TPP copolymer (the ‘T’ in T‐IPIC) as the matrix, and the IPIC with excellent photophysical properties was encapsulated into the NPs via a simple nano‐precipitation method (Figure 5A). Under 808 nm laser irradiation, T‐IPIC NPs exhibited excellent NIR‐II fluorescence signal with a quantum yield (QY) of 2.2% in aqueous solution. The bio tissue penetration of T‐IPIC NPs NIR‐II signal could reach 9 mm. After i.v. injection of T‐IPIC NPs into a HeLa tumor‐bearing mouse model, the vasculature throughout the body was clearly visible. The fluorescence signal at the tumor site reached its peak at 48 hours and gradually declined thereafter. However, even at 96 h, tumor tissue could still be clearly observed, indicating the long‐term imaging capability of T‐IPIC NPs (Figure 5B,C). The PCE of T‐IPIC NPs was determined to be 39.6%, and the singlet oxygen ^1^O_2_ generation efficiency (*Φ*_Δ_) was measured to be 2.3%, which was significantly higher than the clinical NIR dye indocyanine green (ICG, *Φ*_Δ_ 0.2%). These outstanding photophysical properties of T‐IPIC NPs made them suitable for combined PTT and PDT, benefiting from their excellent mitochondrial targeting ability and inherent multifunctionality. Therefore, in the in vivo treatment process, a single‐dose injection and 808 nm laser irradiation alone demonstrated remarkable therapeutic efficacy in HeLa tumor‐bearing mice (Figure 5D–F). This provided a promising approach for the development of new phototherapy systems

TPP derivatives could also be applied in anti‐inflammatory therapy and precise diagnosis. Theranostic agent 1 (TA1) developed by Kim's group comprised four essential components: an aryl thioester for H_2_S_n_‐mediated triggering, TPP for mitochondrial targeting, indomethacin (IMC) as a cyclooxygenase (COX) inhibitor for anti‐inflammatory action, and a Rhodol dye fluorescent reporting group for two‐photon microscopy imaging.^[^ ^6^ ^]^ TA1 exhibited selective accumulation at inflamed regions and underwent a specific reaction with H_2_S_n_ on the mitochondria, triggering a self‐immolation process and concurrent release of indomethacin and Rhodol‐TPP. The self‐immolation process facilitated the opening of the Rhodol moiety, resulting in intense fluorescence emission at the inflammation site (off‐on response). In vivo and *ex vivo* imaging of TA1 in a lipopolysaccharide‐induced acute liver injury mouse model demonstrated prominent fluorescence enhancement signals in the liver. TA1 effectively reached the mitochondria and reacted with the locally generated and stored H_2_S_n_, leading to the liberation of IMC for its anti‐inflammatory effect. Moreover, TA1 significantly reduced COX‐2 expression in cells and diminished the concentration of prostaglandin E_2_ in the serum, both of which were inflammation‐related factors observed in an inflammation‐induced mouse model with H_2_S_n_ overexpression. Therefore, TA1 held great promise as a novel theranostic agent for inflammation management.

#### 4.1.6 Metal–organic complexes

Due to their exceptional photophysical properties and the ease of chemical modification, a plenty of transition metal complexes exhibit promising potential as ideal photosensitizers. Transition metals inherently possess diverse excited states, and their heavy atom effect facilitates fast intersystem crossing (ISC) between singlet and triplet states. The prolonged lifetime resulting from ISC provides favorable conditions for the generation of highly reactive ^1^O_2_ and superoxide radicals.^[^ ^132^ ^]^ The extended emission lifetime exhibited by metal complexes provides a substantial advantage in mitigating self‐fluorescence interference, thus rendering them highly promising in the realm of biosensing and imaging.^[^ ^133^ ^]^

Drawing inspiration from the structural and physicochemical attributes of gemini surfactants, Yi et al. have developed a novel family of amphiphilic gemini iridium(III) complexes (GIC), denoted as Ir1‐Ir6.^[^ ^134^ ^]^ By introducing quaternary ammonium moieties, these GIC compounds exhibited adjustable water solubility and remarkable self‐assembly characteristics, thereby facilitating the spontaneous formation of vesicular structures in aqueous environments. Among the series of Ir1‐Ir6 complexes, Ir3 demonstrated the highest relative QY of singlet oxygen (*Φ*_s_). Furthermore, Ir3 exhibited potent phototoxicity against HepG2 and MCF‐7 cells, with remarkably low half‐inhibitory concentration values of 1.2 µm (phototoxicity index, PI = 250.0) and 1.3 µm (PI = 230.7) respectively. The parameter PI represents the ratio of the half‐inhibitory concentration value under light irradiation compared to that in the absence of light, reflecting the enhanced cytotoxic effect induced by light activation.

Ir3 demonstrated selective enrichment in the mitochondria of tumor cells, exhibiting bright and stable luminescent signals. In in vivo experiments, Ir3 was administered via intratumoral injection at a dosage of 59.5 µg kg^−1^ (25 µL, 40 µm) to tumor‐bearing mice, followed by light exposure (400–700 nm, 250 mW cm^−2^) for 20 min, starting 15 min after injection. The PDT group exhibited significant inhibition of tumor growth, with a gradual reduction in tumor volume over time. In contrast, the control groups showed a notable increase in tumor volume during the same period. Further investigation is required to elucidate the renal clearance behavior of this GIC family.

Liu et al. reported a phosphorescence mitochondria‐accumulating Ru(II) polypyridyl prodrug, RuAzNM, which could be activated under hypoxic conditions.^[^ ^135^ ^]^ RuAzNM was functionalized with both azo and nitrogen mustard (NM) moieties, endowing it with multimodal diagnostic and therapeutic properties against hypoxic cancer cells. The phosphorescence of this prodrug was quenched by photoinduced electron transfer to the azo group, while the electron‐deficient azo group and Ru(II) exhibited an electron‐accepting effect that stabilized and deactivated the NM moiety. In the hypoxic environment of tumor cells, the prodrug accumulated in the mitochondria, where there was an overexpression of reductases such as azoreductase,^[^ ^136^ ^]^ nitroreductase, and DT‐diaphorase. These reductases selectively reduced the azo group, generating the corresponding primary amine products, including the aniline mustard and the parent polypyridyl complex RuNH_2_. The aniline mustard acted as an active alkylating agent, inducing ROS formation and damaging mtDNA, thereby exerting chemotherapy on hypoxic cancer cells. The Ru(II) center exhibited phosphorescence, enabling its application as a probe in hypoxic cells. Furthermore, under directed light excitation, RuAzNM could achieve multimodal therapy, leading to ROS burst, NADH and ATP depletion, mitochondrial membrane potential loss, mtDNA depletion, and ultimately, cell apoptosis.

RuAzNM demonstrated potent anticancer capability against hypoxic HepG2 cells (IC_50_ = 2.3 µm), being tenfold more potent than cisplatin (IC_50_ = 24.5 µm) and twofold more potent than 5‐ALA‐induced protoporphyrin IX (IC_50_ = 6.0 µm). Under normal oxygen conditions, it exhibited 25‐fold higher toxicity towards THL‐3 normal liver cells (IC_50_ = 58.2 µm). Moreover, it exhibited similar phototherapeutic activity (IC_50_ = 3.4 µm) against HepG2 multicellular spheroids with a diameter of approximately 400 µm. These findings suggested that RuAzNM holds promise as an effective hypoxia‐responsive candidate for anticancer drug therapy.

#### 4.1.7 Inorganic nanoparticles

Zheng et al. designed an intelligent nanofactory Ag_2_S:Au@CuS‐GOx/Cel (AUC‐GOx/Cel) that utilized the “external supply, internal promotion” dual‐strategy to amplify intratumoral H_2_O_2_, thereby achieving H_2_O_2_‐activated NIR‐II fluorescence‐guided self‐enhanced chemodynamic and starvation therapy.^[^ ^137^ ^]^ AUC‐GOx/Cel exhibited remarkable NIR‐II fluorescence sensitivity, which could be activated by overexpressed H_2_O_2_ in the TME. GOx, encapsulated within AUC, functioned as a “star” enzyme catalyst, initiating the glucose oxidation process, and generating gluconic acid and hydrogen peroxide. Simultaneously, the loaded celastrol targeted cellular mitochondria to augment endogenous hydrogen peroxide levels. The resulting accumulated H_2_O_2_ not only activated NIR‐II fluorescence but also facilitated a self‐enhanced Cu^2+^‐mediated Fenton‐like reaction, generating hydroxyl radicals that promote synergistic effects between cancer starvation therapy and CDT.

In vivo fluorescence activation imaging experiments demonstrated that the “turn‐on” process of AUC‐GOx/Cel fluorescence remained exclusive to tumor regions, while normal tissues and organs remain unaffected. In H_2_O_2_‐deficient tumor areas, the activatable NIR‐II fluorescence nanofactory, AUC‐GOx/Cel, rapidly and specifically illuminated the tumor site. The fluorescence intensity of AUC‐GOx/Cel within the tumor increased over time. This observation indicated that the GOx‐mediated “external supply” and celastrol‐induced “internal promotion” effectively enhanced intratumoral hydrogen peroxide concentrations, facilitating copper sulfide degradation and promoting the rapid recovery of AUC fluorescence.

Furthermore, the in vivo antitumor activity of AUC‐GOx/Cel was assessed using the 4T1 tumor model. Through the integration of the “external supply” and “internal promotion” strategies, AUC‐GOx/Cel with dual H_2_O_2_‐enhancing capabilities exhibited the most effective tumor suppression after 16 days of treatment. Histological staining with hematoxylin and eosin (H&E) revealed significant apoptotic and necrotic changes in tumor tissues following AUC‐GOx/Cel administration. Mice treated with AUC‐GOx/Cel demonstrated a substantial extension of survival time by 50 days. Notably, AUC‐GOx/Cel exhibited excellent antitumor efficacy and demonstrated favorable biocompatibility, offering new avenues for the design of precision cancer therapeutics.

### 4.2 Theranostic agents for photoacoustic imaging (PAI)

PAI is an emerging imaging technique based on the PA effect that has gained significant popularity in the field of biomedical research in recent years.^[^ ^138^ ^]^ Upon irradiated by nanosecond‐pulsed laser beams, biological samples or probes would generate pulsed localized heating and expansion, leading to the production of acoustic waves which could be detected and imaged by US imaging device.^[^ ^139^ ^]^

Utilizing mechanical waves as imaging signal, PAI shows deeper tissue penetration depth from millimeters to centimeters than other OI methods, such as fluorescence imaging, confocal or two‐photon microscopy, optical coherence tomography, and diffuse optical tomography.^[^ ^140^ ^]^ PAI also offers tunability, allowing for the adjustment of spatial resolution and imaging depth to target specific regions of interest.^[^ ^141^ ^]^ Furthermore, PAI enables functional imaging by quantifying and spatially mapping various biomarkers, including tissue composition, blood flow, metabolism, and neural activity.^[^ ^142^ ^]^

The resolution of PAI tends to decrease with increasing depth due to the scattering of excitation photons in tissues. Additionally, PAI requires complex instrumentation, resulting in relatively higher costs and need for skilled operators with expertise in both optics and US. Nevertheless, PAI remains a promising imaging modality and plays a crucial role in multimodal imaging approaches.

Ji et al. have developed a multifunctional theranostic platform termed PT‐V@TPDOX for the chemo‐photothermal therapy (chemo‐PTT) of multidrug‐resistant breast cancer guided by PAI.^[^ ^143^ ^]^ PT‐V@TPDOX exhibited excellent photothermal effects with a PCE of 42.8%, enabling its use for PTT or temperature‐controlled drug release under laser irradiation. PT‐V@TPDOX effectively delivered triphenylphosphine‐modified doxorubicin (TPDOX) to the mitochondria of MCF‐7/ADR cells, inducing mitochondrial damage and reducing adenosine triphosphate production, thereby inhibiting drug efflux. In vitro experiments demonstrated potent therapeutic efficacy of PT‐V@TPDOX combined with laser irradiation against both the MCF‐7 breast cancer cell line and its multidrug‐resistant counterpart, MCF‐7/ADR cells. These findings suggested that PT‐V@TPDOX can overcome multidrug resistance through enhanced drug retention and combination therapy.

PT‐V@TPDOX solution (200 µL, 200 µg mL^−1^) was intravenously injected into female BALB/c mice carrying MCF‐7/ADR tumors, resulting in observable PA signals at the tumor site that reached maximum intensity at 12 h. Benefitting from the dual effects of enhanced drug retention and chemo‐PTT combination therapy, PT‐V@TPDOX combined with laser irradiation demonstrated remarkable antitumor capability against BALB/c mice bearing MCF‐7/ADR tumors, achieving a tumor inhibition efficiency of 97.3%. The successful implementation of PA‐guided chemo‐PTT using PT‐V@TPDOX represented an effective strategy to overcome multidrug resistance.

### 4.3 Theranostic agents for MRI

MRI signals are generated by the process of nuclear magnetic relaxation of excited hydrogen nuclei within the human body induced by radiofrequency pulses.^[^ ^144^ ^]^ Compared to conventional X‐ray imaging techniques, MRI does not involve ionizing radiation, rendering it a safer modality and particularly suitable for individuals requiring repetitive imaging procedures. MRI exhibits exceptional tissue differentiation capabilities, enabling the visualization of intricate anatomical structures and pathological alterations, thereby providing accurate diagnostic information.^[^ ^145^ ^]^ In clinical practice, MRI finds applications in distinguishing between benign and malignant tumors, quantitatively assessing microstructural characteristics of the brain in both healthy and diseased individuals, evaluating cardiac morphology and ventricular function, diagnosing spinal injuries, and more.^[^ ^144^, ^146^ ^]^

Nonetheless, MRI technology possesses certain limitations, such as its relatively high cost, extended examination duration that may be inconvenient for emergency cases, susceptibility to artifacts that can complicate image interpretation, and limited availability in certain settings.^[^ ^147^ ^]^ Furthermore, as MRI generates a strong magnetic field during the imaging process, precautions must be taken to ensure that individuals within the examination room do not carry ferromagnetic materials

Patra et al. employed the chemotherapeutic agent camptothecin (CPT), a mitochondria‐targeting moiety (TPP cation), a receptor‐targeting ligand (biotin), and an MRI contrast agent (iron complex) to prepare a sequential receptor‐mitochondria dual targeting polyprodrug, CP TP PG BN Fe, for potential chemotherapy and simultaneous MRI diagnosis.^[^ ^148^ ^]^ This amphiphilic polyprodrug spontaneously assembled into nanospheres, exhibiting significant T1‐weighted MRI contrast enhancement. The longitudinal and transverse relaxation constants were meticulously evaluated, yielding the following values: r1 = 19.43 mm ^−1^·s^−1^ and r2 = 67.48 mm ^−1^·s^−1^. Moreover, the ratio of r2 to r1 (r2/r1 = 3.47) demonstrated the efficiency of the therapeutic polyprodrug, substantiating the effectiveness of CP TP PG BN Fe as a potential T1 contrast agent for MRI. Spectral demonstrations of longitudinal (T1) relaxation and transverse (T2) relaxation indicated the potential of the therapeutic polyprodrug CP TP PG BN Fe as a promising MRI‐active agent.

In vitro cellular experiments revealed that the therapeutic polyprodrug CP TP PG BN Fe possessed superior dual‐targeting capabilities for continuous receptor and mitochondria targeting compared to the control group (CP PG BN). It enabled efficient and precise delivery of CPT into cancer cells. In comparison to free CPT, the therapeutic polyprodrug CP TP PG BN Fe exhibited heightened activities in terms of mitochondria‐targeting, cellular cytotoxicity, apoptosis induction, mitochondrial ROS amplification, caspase‐3 activation, and mitochondrial depolarization. Through a sequential receptor and mitochondria dual‐targeting strategy, CP TP PG BN Fe demonstrated the potential for effective and precise delivery of mitochondrial CPT, thereby presenting a prospective approach for cancer treatment. However, further experiments were required to evaluate its in vivo therapeutic efficacy.

### 4.4 Multimodality imaging and its use in mitochondria‐targeting theranostic

Multimodal imaging can compensate for the limitations of single‐modal technics, providing additional diagnostic information. For instance, MRI possesses the capability to differentiate soft‐tissues with high resolution, allowing for the visualization of complex anatomical structures and pathological changes. However, it rarely offers biochemical and metabolic information, which can be supplemented by PET.^[^ ^149^ ^]^

The affordability, real‐time capabilities, and the variety of modifiable structures make optical imaging widely applicable in multimodal biomedical research.^[^ ^150^ ^]^ However, limited light penetration and light scattering in bio‐tissues obstacles the obtain of quantitative or tomographic information. Therefore, in scenarios that require deep tissue imaging, cooperating with other modalities such as nuclear imaging, ultrasound (US), and MRI becomes particularly important.^[^ ^151^ ^]^ Many photoresponsive strategies impart numerous distinctive advantages to phototheranostics, such as spatiotemporal control of drug release and biological imaging.^[^ ^152^ ^]^ Combining nuclear imaging techniques with optical imaging methods will enhance the precision of phototheranostics, offering an excellent perspective for the precise treatment of cancers. There is still a need to further enrich small molecules with multi‐modal imaging functionalities.

Multimodal theranostic agents are valuable for comprehensive disease assessment, but they may be considered unnecessary when single‐modal imaging or therapy suffices for theranostic purposes. Furthermore, integrating multimodal theranostics into small molecules could significantly alter their biodistribution and in vivo pharmacokinetics, potentially reducing their accumulation at plaque sites or causing unwanted off‐target toxicity. In the process of constructing multimodal molecules, certain conditions such as high temperatures or acidity/alkalinity in radionuclide labeling process before PET imaging can limit the applicability of many optical probes. Balancing the different administration dosage between different modalities to maintain proper imaging quality is another challenge in multi‐modal theranostics.

Various works have combined the strengths of different imaging techniques to provide enhanced imaging capabilities and a more comprehensive evaluation of the targeted tissues or organs, ultimately improving the overall diagnostic accuracy and clinical outcomes.

#### 4.4.1 PAI and OI agents

**FIGURE 6:**
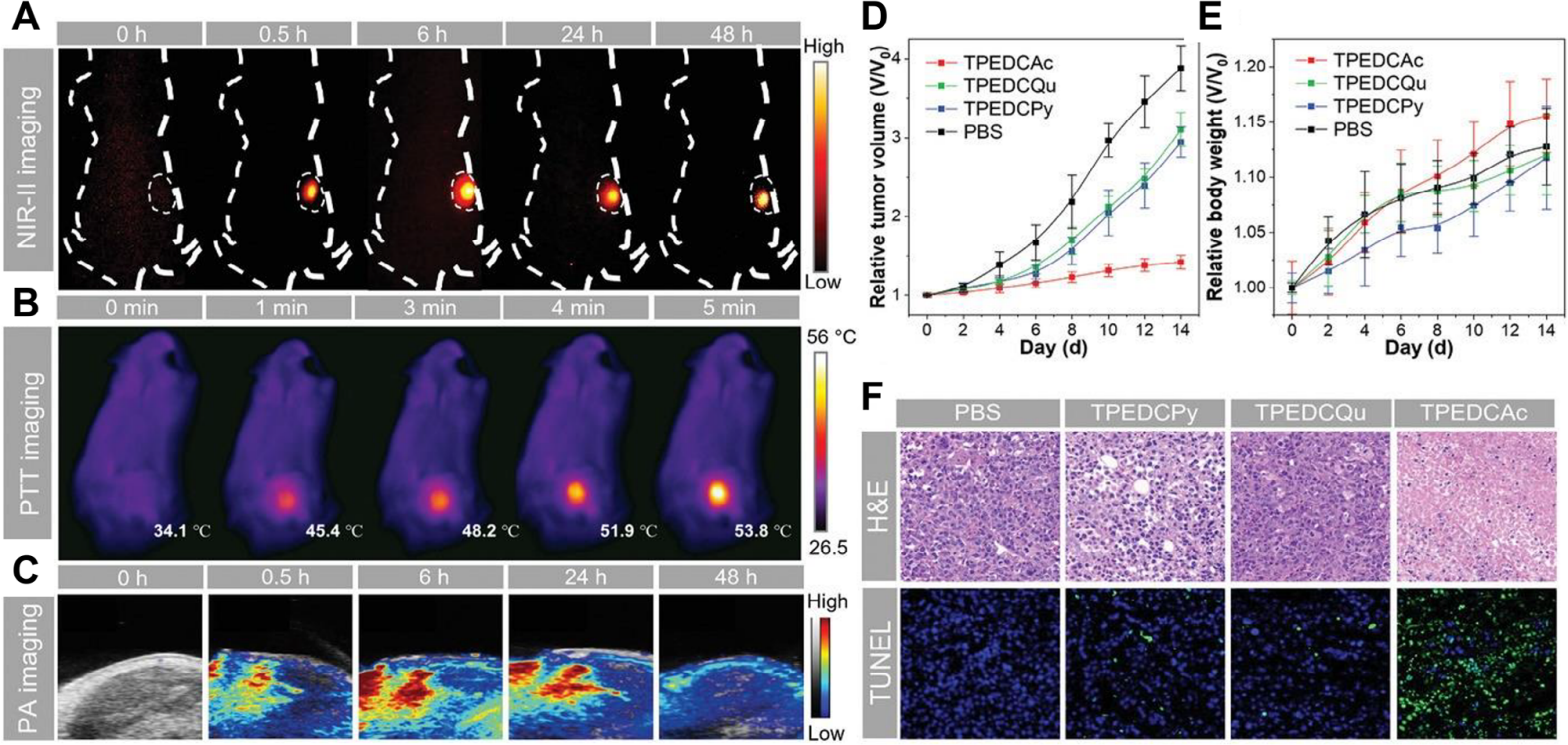
AIEgens capable of multimodal imaging and therapy. (A) NIR‐II fluorescence imaging of the mice at different time after intratumoral administration of TPEDCAc aggregates under laser irradiation (660 nm, 0.3 W cm^−2^). (B) Infrared thermal images of MCF‐7 tumor‐bearing mice intratumorally injected with TPEDCAc aggregates followed by (12 h later) laser irradiation (660 nm, 0.3 W cm^−2^) for 1, 3, 4, and 5 min. (C) PA imaging of MCF‐7 tumor‐bearing mice after intratumoral injection of TPEDCAc aggregates (1 × 10^−3^ m, 20 µL). (D) Growth curves of the xenografted MCF‐7 tumors on mice (*n* = 5) after treatment with PBS alone, TPEDCPy, TPEDCQu, and TPEDCAc aggregates (1 × 10^−3^ m, 20 µL) and laser irradiation. (E) Body weight curves of the xenografted MCF‐7 tumor‐bearing mice after treatment as described in (D). (F) Hematoxylin and eosin (H&E) and TUNEL staining analyses of tumor tissues treated with various AIEgens. The TUNEL cells are stained in green emission. Reproduced with permission.^[^ ^153^ ^]^ Copyright 2022, Wiley‐VCH GmbH.

As mentioned previously *N*‐alkylpyridinium based structure showed good PTT ability and therefore had high potential in PAI. Zhang et al. employed an electron acceptor engineering strategy to regulate intramolecular motion and construct D‐A type AIE phototheranostic agents with mitochondria‐targeting capability, achieving multimodal synergistic phototherapy guided by NIR‐II/PA imaging.^[^ ^153^ ^]^ Among the prepared analogs (TPEDCPy, TPEDCQu, and TPEDCAc), TPEDCAc, which exhibited the strongest D‐A intensity and the largest acceptor rotor, emerged as the most promising multimodal phototherapeutic agent. TPEDCAc demonstrated a broad redshifted absorption profile, displaying high absorption even at longer wavelengths such as 660 nm. It exhibited an NIR emission peak at 980 nm, with a substantial portion falling within the NIR‐II region. The enhanced ICT intensity and redshifted emission resulted in a relatively low QY of 0.4%. In vivo NIR‐II fluorescence imaging was performed by intratumoral injection of TPEDCAc into MCF‐7 tumor‐bearing mice. Under 660 nm laser excitation, strong fluorescence signals were observed at 6 h, and even after 48 h, the tumor remained clearly visible, indicating effective long‐term tumor tracking capability (Figure 6A). Photothermal imaging monitored the temperature increase at the tumor site from 34.1 to 53.8°C upon irradiation with a 660 nm laser at a power density of 0.3 W cm^−^^2^ (Figure 6B). Furthermore, the PA signal in the tumor region persisted for up to 24 hours post‐injection (Figure 6C).

TPEDCAc exhibited a mixture of Type I and Type II ROS generation, with a calculated PCE of TPEDCAc aggregates reaching 44.8%. After 10 min of 660 nm laser irradiation, a concentration of 15 µg mL^−1^ TPEDCAc resulted in the killing of >90% of MCF‐7 cancer cells. In in vivo antitumor experiments conducted on MCF‐7 tumor‐bearing nude mice, significant suppression of tumor growth was observed under the synergistic PDT and PTT effects of TPEDCAc (Figure 6D–F). The design principle of TPEDCAc provides an effective strategy for the rational design of next‐generation imaging‐guided phototherapeutic agents.

By introducing an extra *N*‐alkylpyridinium and using supramolecular strategy, Song et al. fabricated a carboxylatopillar[5]arene (CP5)‐modified multifunctional theranostic nanosystem, denoted as TTPY‐Py⊂CP5@AuNR.^[^ ^154^ ^]^ The TTPY‐Py molecule, bearing positively charged pyridinium groups, served as an AIE active photosensitizer and exhibited targeted mitochondria delivery capability. Cetyltrimethylammonium bromide (CTAB) was usually utilized for the stabilization of gold nanorods (AuNRs). However, it posed inherent biotoxicity concerns. Substituting CTAB with CP5 enabled improved phototherapeutic and diagnostic properties of AuNRs within the biological matrix. The encapsulation and release of TTPY‐Py in TTPY‐Py⊂CP5@AuNR nanosystem could be precisely controlled under the influence of three stimuli, including pH, temperature, and NIR irradiation.

The TTPY‐Py molecule encapsulated within TTPY‐Py⊂CP5@AuNR nanosystem could efficiently be internalized by cells and, upon exposure to the acidic environment of lysosomes (pH = 5.0–5.5), trigger the release of TTPY‐Py through the modulation of host‐guest interactions, resulting in specific fluorescence activation within the mitochondria region. Furthermore, this release process could also be activated by the localized temperature increase induced by 808 nm laser irradiation. The TTPY‐Py⊂CP5@AuNR nanosystem demonstrated excellent fluorescence imaging and PAI capabilities in vivo, facilitating accurate tumor identification and real‐time imaging for therapeutic monitoring. Although the ROS generation capacity of TTPY‐Py⊂CP5@AuNR nanosystem was relatively lower than that of pure TTPY‐Py, its photothermal capability enabled synergistic therapeutic effects. The PCE under 1.0 W cm^−^^2^ irradiation was approximately 30.0%. Notably, the combination of PTT and PDT mediated by TTPY‐Py⊂CP5@AuNR nanosystem achieved complete tumor growth suppression without recurrence under white light and 808 nm NIR laser irradiation. Moreover, the presence of CD31‐positive blood vessel formation indicated the effective inhibition of tumor neovascularization. The development of hybrid nanomaterials through the supramolecular approach offered significant potential for advancing multifunctional phototheranostic agents.

Shin et al. designed a mitochondria‐targeting donor‐acceptor‐donor (D‐A‐D) molecular structure, MsPDTT, as an effective photosensitizer for cancer PDT and PTT. For a biocompatible formulation, this structure was encapsulated into Food and Drug Administration (FDA)‐approved amphiphilic copolymers (DSPE‐mPEG5000) to form MsPDTT NPs.^[^ ^155^ ^]^ Excited at 690 nm, MsPDTT NPs enabled dual‐modal PA and NIR fluorescence imaging, as well as efficient PDT/PTT. RsPDTT NPs were developed as a control counterpart, lacking mitochondria‐targeting groups (TPP) and showing only PTT effects.

**FIGURE 7:**
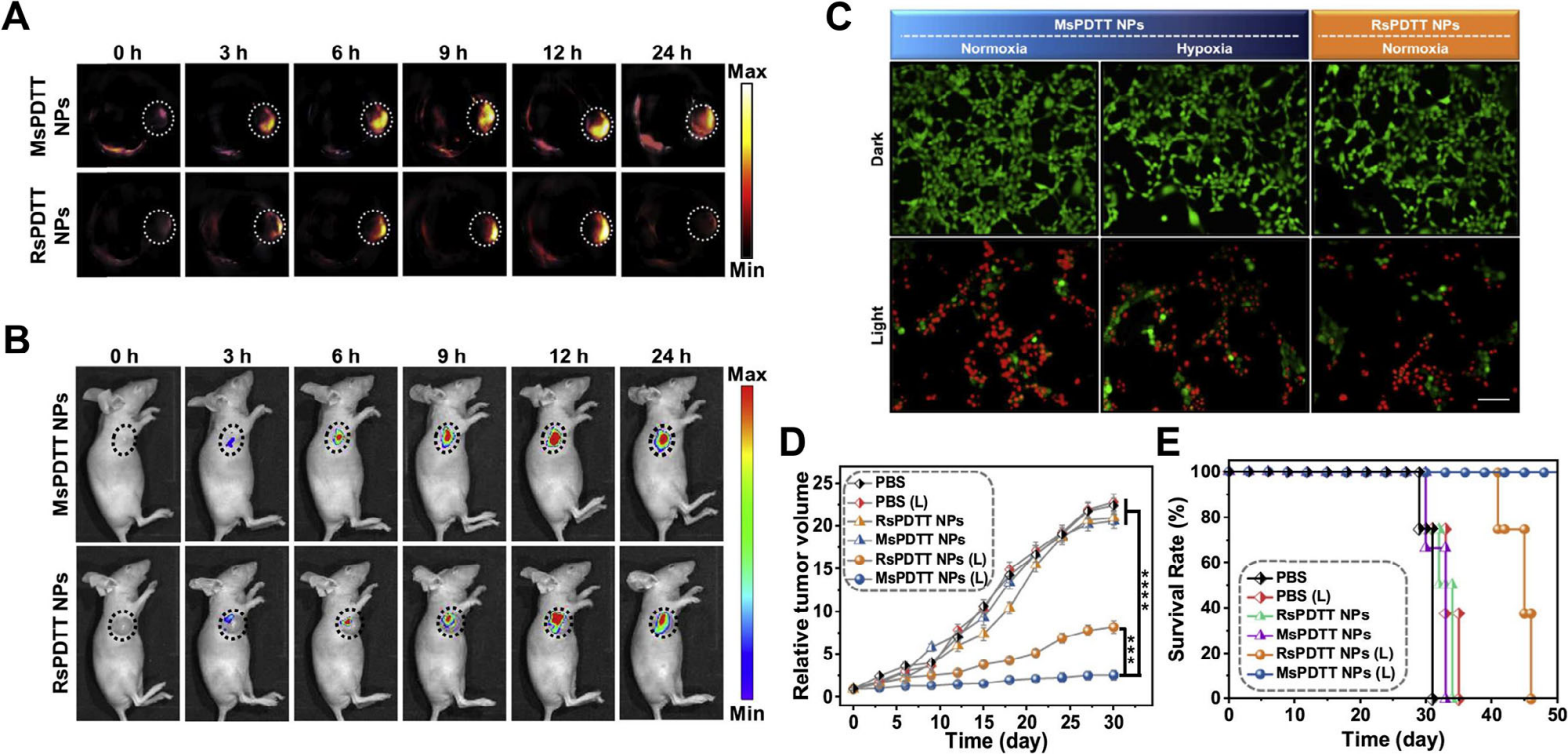
Effective photosensitizer MsPDTT for cancer phototherapy. (A) PA imaging of 4T1 tumors (0, 3, 6, 9, 12, and 24 h) after i.v. injection of MsPDTT NPs and RsPDTT NPs. (B) Fluorescence imaging of 4T1 tumor‐bearing mice after i.v. injection of MsPDTT NPs and RsPDTT NPs (150 µm, 200 µL). (C) Images of calcein‐AM‐ and PI‐labeled, MsPDTT NP‐ (10 µm) treated 4T1 cells under normoxic (21% O_2_) and hypoxic conditions (0.1% O_2_, 5% CO_2_) with or without 690 nm laser irradiation (1.0 W cm^−2^, 10 min) and RsPDTT NP‐ (10 µm) treated 4T1 cells under normoxic conditions (21% O_2_) with or without light irradiation. Scale bar, 100 µm. (D) Relative tumor volumes of mice after different treatments. (E) Survival rates of the mice in each tested group after different treatments (*n* = 3). For all tests, significance is defined as ∗∗∗∗*p* < 0.0001, ∗∗∗*p* < 0.001, ∗∗*p* < 0.01; *p* > 0.05 was considered not statistically significant (n.s.). Reproduced with permission.^[^ ^155^ ^]^ Copyright 2021, Elsevier.

Semi‐quantitative analysis of PA signals and fluorescence intensity after i.v. administration of these agents to 4T1 tumor‐bearing mouse model indicated consistent changes in the dual‐modal imaging signals. Both MsPDTT NPs and RsPDTT NPs gradually accumulated at the tumor site and maximized at 12 h post‐injection (Figure 7A,B). The incorporation of TPP cations into the MsPDTT framework enhanced not only the mitochondrial targeting capability of the prepared NPs but also endowed them with dual phototherapeutic properties. MsPDTT NPs, under laser irradiation in solution, generated both heat and ROS, making them suitable candidates for PTT and PDT. MsPDTT NPs exhibited effective anticancer activity under both hypoxic and normoxic conditions, suggesting the advantage of dual phototherapy for light‐induced treatment. The investigation of PDT‐ or PTT‐mediated cytotoxicity revealed that the anticancer activity of MsPDTT NPs under hypoxic conditions was primarily attributed to the PTT effect, while both PTT and PDT showed killing effects on tumor cells under normoxic conditions, with a greater contribution from PTT (Figure 7C). For animal model treatment experiment, only mice receiving i.v. injection of MsPDTT NPs and laser irradiation exhibited a significantly extended lifespan of 50 days (Figure 7D,E). These results suggested that combining PDT and PTT to maximize photon utilization could help overcome the resistance of traditional PDT‐related hypoxia‐mediated tumor photodamage, leading to significant inhibition of tumor growth and enhanced therapeutic effects.

Gold nanorods (Au NRs) are excellent contrast agents for PAI and PTT.^[^ ^156^ ^]^ However, their high toxicity, poor biocompatibility, rapid clearance, and dependence on external laser sources limit their applications. Zhang and colleagues modified gold nanorods (Au NRs) with carbon‐based nanomaterials to create a multifunctional theranostic nanomaterial called mdGC, capable of dual‐mode imaging using PA and fluorescence.^[^ ^157^ ^]^ This material reduced the side effects associated with Au NRs and demonstrated improved biocompatibility, enhanced cell toxicity, precise imaging capabilities, and targeted delivery without the need for external light source excitation. It effectively overcame the heat‐related issues caused by laser use in traditional PTT.

Bio‐TEM images revealed the internalization of mdGC into 4T1 cells and its localization within the mitochondria. The treated cells showed a decrease in mitochondrial area and perimeter, and the mitochondrial cristae nearly disappeared, indicating the disruption of mitochondrial morphology. mdGC exhibited superior PAI performance compared to Au NRs, with the highest PA signal observed at 2 h, consistent with the fluorescence imaging results. After a 2‐h injection, mdGC accumulated at the tumor site with an accumulation amount of gold (IDg^−1^) of 20.71%, while the Au NR group only reached 3.78% IDg^−1^.

To investigate the in vivo anti‐tumor capability, mdGC (300 mg L^−1^) and Au NRs (300 mg L^−1^) were administered through the tail vein. Mice in the mdGC group exhibited good overall conditions, and based on the relative tumor volume calculation, the tumor growth inhibition rate was as high as 80.44%. However, the Au NR group gradually experienced adverse reactions, such as fatigue and anorexia, leading to mortality starting on the 14th day after treatment. Unlike Au NRs, mdGC exerted its anti‐tumor effect not through PTT, but by increasing intracellular ROS production, reducing MMP, and modulating the expression of apoptosis‐related genes. Notably, mdGC significantly decreased the expression of cell proliferation‐related genes, such as c‐Myc and cyclin D1, through the induction of mitochondrial‐mediated cell apoptosis. mdGC induced cell apoptosis through the mitochondrial pathway, thereby achieving its anti‐tumor effects.

Cellular energy in the form of ATP plays a pivotal role in various processes involved in DNA damage recognition, DNA binding, DNA double‐strand unwinding, and eventual DNA damage excision and repair.^[^ ^158^ ^]^ Zhang et al. developed a novel theranostic agent, namely IR780@Pt NPs, by integrating a cisplatin (CDDP) prodrug (Pt‐CD) and a mitochondria‐targeting NIR photosensitizer, IR780, using a supramolecular approach.^[^ ^159^ ^]^ This multifunctional nanodrug was designed to synergistically enhance the anticancer effect of CDDP by disrupting the energy supply to the nucleotide excision repair (NER) pathway through mitochondrial dysfunction. Upon i.v. administration of IR780@NPs in mice bearing 143B tumors, the NIR fluorescence intensity gradually increased at the tumor site, peaking at 24 h. PAI confirmed the time‐dependent tumor‐targeting properties of IR780@Pt NPs. Subsequent laser irradiation (808 nm, 1 W cm^−2^, 5 min) of the 143B tumor‐bearing mice treated with IR780@Pt NPs resulted in a rapid temperature elevation at the tumor site, reaching 59.5 ± 2.6°C, thereby significantly inducing tumor ablation. Following a 14‐day treatment period, the tumor volume in the mice decreased from 150 to 52.8 mm^3^. Moreover, IR780@Pt NPs downregulated the expression of key components involved in the NER pathway, including excision repair cross‐complementing‐1 and xeroderma pigmentosum complementation group F, hence further enhancing the anticancer efficacy of CDDP.^[^ ^158^, ^160^ ^]^ The mitochondria‐targeted theranostic nanomedicine, IR780@Pt NPs, exemplifies the potential of multifunctional nanomaterials in enhancing cancer treatment synergistically.

#### 4.4.2 Fluorescent MRI probe for theranostic

Wen et al. developed a degradable carrier‐free curcumin‐based metal‐phenolic network theranostic agent with targeted mitochondrial damage, namely ICG loaded curcumin‐Gd nanoparticle (ICG@cur‐Gd NP), for MRI/fluorescence dual‐modal imaging‐guided chemotherapy/PDT combined cancer treatment.^[^ ^161^ ^]^ Curcumin was dissolved in dimethyl sulfoxide and combined with Gd^3+^ to form a curcumin‐Gd^3+^ complex, which self‐assembled into cur‐Gd NPs in an ethanol solution. ICG was then loaded onto the cur‐NPs through electrostatic attraction to obtain ICG@cur‐Gd NPs, which exhibited good water solubility and green fluorescence. Curcumin induced mitochondrial damage and cancer cell death. The integration of curcumin, Gd^3+^, and ICG in ICG@cur‐Gd NPs allowed MR/fluorescence imaging‐guided chemotherapy/PDT.

**FIGURE 8:**
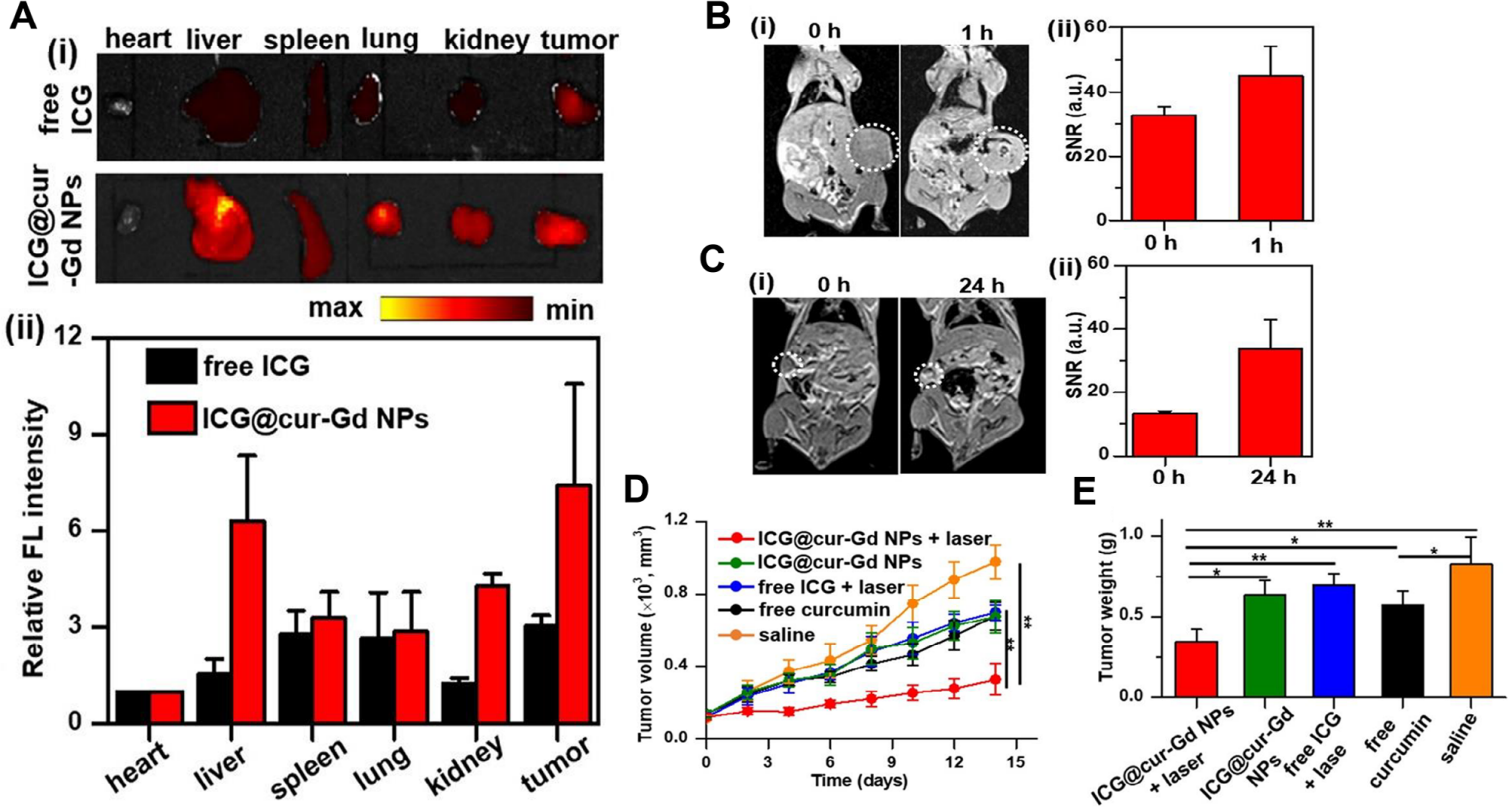
Metal‐phenolic network theranostic agent for MRI, fluorescence imaging, and cancer treatment. (A‐i) Ex vivo fluorescence images and (ii) average fluorescence intensity of major organs and tumors excised from tumor‐bearing mice at 24 h post‐treatment. (B,C‐i) In vivo MRI and (ii) quantitative magnetic resonance signal‐to‐noise ratio (SNR) of tumor‐bearing mice (B) at 0 and 1 h after the intratumoral administration of ICG@cur‐Gd NPs and (C) at 0 and 24 h after the intravenous administration of ICG@cur‐Gd NPs. 0 h means the pretreatment of the mice. (D) Tumor growth curves of tumor‐bearing mice after various treatments. (E) Weights of excised tumors from tumor‐bearing mice at day 14 after various treatments. Reproduced with permission.^[^ ^161^ ^]^ Copyright 2021, American Chemical Society.

The dual‐modal MRI/fluorescence imaging of ICG@cur‐Gd NPs provided accurate tumor visualization for guiding chemotherapy/PDT in cancer treatment. In vivo fluorescence imaging demonstrated enhanced accumulation of ICG@cur‐Gd NPs at the tumor site due to the enhanced permeability and retention (EPR) effect, leading to prolonged retention time within the tumor. Ex vivo fluorescence quantification analysis showed approximately 2.5‐fold higher accumulation of ICG@cur‐Gd NPs in the tumor compared to free ICG after 24 h of treatment (Figure 8A). The calculated longitudinal relaxivity (r1) in T1‐weighted MR imaging capability was 4.66 mm ^−1^ s^−1^, indicating that ICG@cur‐Gd NPs are suitable as T1 MR imaging contrast agents.^[^ ^162^ ^]^ In in vivo MRI, the corresponding SNR increased by 1.4‐fold at 1 hour post‐intratumoral treatment (Figure 8B) and 2.5‐fold at 24 h (Figure 8C) post‐i.v. treatment compared to the pre‐treatment level. This indicated that ICG@cur‐Gd NPs can serve as an effective fluorescence/MR imaging contrast agent.

The degradation of ICG@cur‐Gd NPs induced by the tumor acidic environment contributed to tumor treatment and enhanced specificity and biocompatibility. Compared to free ICG, ICG@cur‐Gd NPs exhibited higher ^1^O_2_ generation efficiency. Laser irradiation enhanced the degradation of ICG@cur‐Gd NPs, resulting in the release of free curcumin and subsequent mitochondrial damage in cells. In vivo anti‐tumor experimental results demonstrated significant tumor volume reduction in mice treated with ICG@cur‐Gd NPs plus laser irradiation compared to those treated with saline, free curcumin, or free ICG plus laser irradiation, indicating effective tumor growth inhibition (Figure 8D,E). Histopathological analysis of major organs showed no significant abnormalities, and blood biochemistry analysis revealed that levels of aspartate transaminase, alanine transaminase, creatinine, and blood urea nitrogen remained within the normal range after treatment with saline, free curcumin, free ICG plus laser irradiation, ICG+NPs, or ICG plus laser irradiation. However, similar to most nanomaterials, ICG@cur‐Gd NPs exhibited liver accumulation compared to free ICG, requiring further investigation regarding their biocompatibility.

#### 4.4.3 Nanoplatform integrated Raman imaging, PAI, and fluorescence imaging

Gold nanomaterials exhibit a multitude of captivating properties, including a significantly large surface area‐to‐volume ratio, distinctive optical and electronic features such as robust localized surface plasmon resonance, distance‐dependent optical properties, exceptional biocompatibility, and facile functionalization with molecules such as antibodies, peptides, and nucleic acids to yield functional derivatives.^[^ ^163^ ^]^ These attributes render gold nanomaterials highly appealing for a wide range of academic and scientific applications.

Sun et al. integrated fluorescence/PA/surface‐enhanced Raman imaging with NIR‐II PTT and NIR‐I PDT into a single system, yielding a novel multifunctional nanotheranostic agent called AuND‐TPP‐ICG@MCM.^[^ ^164^ ^]^ Specifically, gold nanodendrites (AuND) were engineered to exhibit absorption from NIR‐I to NIR‐II by modulating the geometric morphology of dendritic branches, thus enabling PTT effects. To evade immune surveillance by the mononuclear phagocyte system and enhance active targeting towards MDA‐MB‐231 cells, the gold nanomaterial was coated with macrophage cell membrane (MCM). Additionally, the triphenylphosphonium moiety was incorporated to provide mitochondrial targeting capability and serve as a reporter gene for surface‐enhanced Raman scattering (SERS) imaging. ICG was utilized as a photosensitizer to enable NIR‐I PDT and fluorescence imaging.

In vitro experiments demonstrated high uptake efficiency of AuND‐TPP‐ICG@MCM by MDA‐MB‐231 cells, and the distribution of AuND‐TPP‐ICG@MCM within MDA‐MB‐231 cells was directly observed through SERS imaging. Under continuous laser irradiation (1064 nm + 808 nm), AuND‐TPP‐ICG@MCM exhibited the highest killing efficiency against MDA‐MB‐231 cells through the combined effects of PTT and PDT. The incorporation of MCM coating and TPP modification effectively enhanced selectivity towards tumor tissues and mitochondrial targeting capability, thereby further augmenting the synergistic therapeutic effects of PDT and PTT. In in vivo experiments, AuND‐TPP‐ICG@MCM displayed superior blood retention compared to AuND‐TPP‐ICG@PEG, and fluorescence signals indicated preferential accumulation in tumor regions. The PA signal peaked at 12 h post‐injection and gradually decreased thereafter. Using MDA‐MB‐231 tumor‐bearing mice, the in vivo anti‐tumor efficacy of AuND‐TPP‐ICG@MCM was investigated, and with laser irradiation at 1064 nm and 808 nm, the tumor almost completely disappeared after 16 days. Furthermore, PDT exhibited slightly stronger anti‐tumor effects than PTT during the treatment process. AuND‐TPP‐ICG@MCM demonstrated promising clinical applications due to its biocompatibility and integration of multimodal diagnosis and therapy capabilities.

#### 4.4.4 Integration of PAI, fluorescence imaging, and US imaging

Wang et al. engineered a novel sequentially targeted theranostic nanoplatform, IR825@Bev‐PLGA‐PFP NPs (IR825@B‐PPNs), for the diagnosis/monitoring and synergistic antiangiogenic PTT of anaplastic thyroid carcinoma (ATC) by multimodal imaging guidance.^[^ ^165^ ^]^ IR825 effectively provided PA and fluorescence imaging capabilities, while perfluoropentane (PFP) offered US imaging ability under NIR irradiation. In vitro targeting experiments illustrated that IR825@B‐PPNs exhibited excellent sequential targeting capabilities. Bevacizumab (Bev) is an FDA‐approved anti‐VEGF monoclonal antibody with a strong affinity for VEGF.^[^ ^166^ ^]^ It imparted specific VEGF targeting and antiangiogenic capabilities to the NPs, allowing them to locate the surface of cells overexpressing VGFR. Subsequently, the NPs selectively accumulated in subcellular organelles, specifically the mitochondria, through the action of IR825.

To investigate the in vivo multimodal imaging capabilities, C643 tumor‐bearing mice were intravenously injected with IR825@B‐PPNs. The PA signal reached its peak at 3 h and persisted for 24 h, while US signals within the tumor area were obtained by laser irradiation for 5 min (0.8 W cm^−2^) after 3 h of i.v. injection. Strong fluorescence signals concentrated at the central position of the tumor area, reaching their peak at 3 h, consistent with other imaging results. The multimodal imaging results demonstrated satisfactory in vivo targeting capabilities of IR825@B‐PPNs carrying IR825 and Bev, allowing effective enrichment at the tumor site and providing detailed diagnostic and real‐time monitoring information for treatment. Similarly, in vivo anti‐tumor experiments were conducted using C643 tumor‐bearing mice. Based on the imaging results, laser irradiation (0.8 W cm^−2^, 10 min) was performed after 3 h of IR825@B‐PPNs injection, resulting in an instantaneous temperature increase to 54.7°C within 30 s in the tumor region. After 2 min of irradiation, the temperature remained at 57°C, achieving a highly satisfactory tumor ablation effect. Necrosis of the tumor area occurred 2 days later, and complete eradication of the tumor was observed after 18 days, with no recurrence observed during continuous observation for 30 days. IR825@B‐PPNs, a smart and multifunctional therapeutic nanoplatform with a simple core/shell spherical structure, improved the cascade accumulation of NPs at the tumor site through precise sequential targeting, enabling synergistic antiangiogenic PTT against ATC. This strategy provided innovative guidance for achieving precise diagnosis and therapy integration of ATC and further clinical translation.

#### 4.4.5 Theranostic materials capable of PAI, fluorescence imaging, and MRI

Typically, nanoparticles need to undergo “CAPIR cascade” to be delivered to solid tumors, which involves circulation in the bloodstream, accumulation through leakage in the tumor, subsequent penetration into the deep regions of the tumor tissue to reach tumor cells, internalization into these cells, and finally drug release within the cells.^[^ ^167^ ^]^ Hierarchical targeting is a novel stimulus‐responsive targeting strategy that converts negative charges to positive charges, enabling high tumor tissue accumulation, efficient cellular internalization, and deep penetration, thereby enhancing the therapeutic efficacy of cancer nanomedicine.^[^ ^168^ ^]^ The extracellular pH can be effectively acidified by releasing Fe(III), significantly improving the efficiency of hierarchical targeting and achieving “CAPIR.”

Liang *et al*. designed a cascade hierarchical targeting theranostic integrated strategy for hypoxic tumors, using Shuttle‐like Fe‐doped polydiaminopyridine nanofusiforms as a type of theranostic ion organic‐inorganic hybrid material.^[^ ^169^ ^]^ These nanofusiforms were loaded with a thermal decomposition radical generator (AIPH) and a photothermal agent (ICG), constructing a mitochondria‐depleting nanoshuttle for precise cancer treatment through oxygen‐independent radical generation guided by multimodal imaging. AIPH/ICG@Fe‐PDAP remained negatively charged in the physiological environment (pH 7.4), but converted to a positive charge under weakly acidic conditions in the TME, enabling hierarchical targeting functionality. The released iron facilitated accelerated cellular uptake and promoted deep penetration, thereby enhancing hierarchical targeting efficiency.

AIPH/ICG@Fe‐PDAP exhibited strong NIR absorption, MRI, and NIR fluorescence imaging capabilities, and could serve as a contrast agent to enhance PAI. Intravenous injection of AIPH/ICG@Fe‐PDAP suspension resulted in the strongest MR signal at the tumor site after 6 h, consistent with PAI results. The loading of ICG endowed AIPH/ICG@Fe‐PDAP with in vivo fluorescence imaging capability. Due to its enhanced EPR effect, AIPH/ICG@Fe‐PDAP gradually accumulated in the tumor region and exhibited the strongest fluorescence signal at 6 h post‐injection. Overall imaging results signified that 6 h was the optimal treatment window. AIPH/ICG@Fe‐PDAP could target mitochondria in tumor cells, effectively depleting glutathione (GSH) in tumor cells to enhance free radical therapy and induce cell apoptosis, demonstrating excellent anti‐tumor efficacy in vivo in 4T1 tumor‐bearing nude mice with a high tumor inhibition rate (>95%). This work has important implications for exploring multimodal imaging theranostic probes for precise treatment of solid hypoxic tumors.

#### 4.4.6 PAI, MRI, US imaging and therapeutic effect in one particle

In addition to its application in tumor therapy, mitochondrial‐targeted therapeutics can also be utilized for suppressing plaque angiogenesis to prevent adverse cardiovascular and cerebrovascular events associated with atherosclerosis.

Yao et al. successfully fabricated a safe, low‐intensity focused US (LIFU)‐responsive, mitochondrial‐targeted, multifunctional nanoplatform called PFP‐HMME@PLGA/MnFe_2_O_4_‐Ram nanoparticles (PHPMR NPs) by encapsulating 3 nm manganese ferrite (MnFe_2_O_4_), hematoporphyrin monomethyl ether (HMME), and PFP in PLGA and modifying the surface with ramucirumab (Ram).^[^ ^170^ ^]^ Ram provided PHPMR NPs with cell‐targeting capability, enabling them to be absorbed by rabbit aortic endothelial cells (RAECs) through active antigen‐antibody interaction. PHPMR NPs could accumulate in the mitochondria of RAECs and exert SDT by inducing ROS‐mediated mitochondrial‐caspase apoptosis. In vivo MRI imaging results showed that after i.v. injection of PFP‐HMME@PLGA/MnFe_2_O_4_‐Ram NPs (1 mg mL^−1^, 50 mL, Fe+Mn: 269.7 mg L^−1^), plaques were clearly observed, and the signal reached its peak 90 min after injection. Compared to Gd‐based contrast agents, MnFe_2_O_4_‐loaded targeted NPs not only exhibited a stronger MRI T1 imaging effect but also had a longer duration of accumulation in the plaques, making them suitable as MRI contrast agents. The addition of MnFe_2_O_4_ significantly enhanced the PAI effect of PHPMR NPs. Following the i.v. injection of PFP‐HMME@PLGA/MnFe_2_O_4_‐Ram NPs into plaque‐bearing rabbits, the PA signal in the plaque region gradually increased and reached its peak at 90 min. PHPMR NPs manifested excellent active targeting behavior toward neovessels in the plaques, making them an ideal PAI contrast agent. Furthermore, experimental evidence confirmed that PFP encapsulated in NPs could generate microbubbles in situ during LIFU treatment, allowing it to serve as a contrast agent for US imaging.

SDT mediated by PFP‐HMME@PLGA/MnFe_2_O_4_‐Ram NPs could effectively inhibit neovascularization and increase the stability of late‐stage atherosclerotic plaques in rabbits. On the 3rd day post‐treatment, the number of apoptotic neovessels increased by approximately 1.35 times, and the hypoxic area was reduced by 50% compared to the control group. On the 28th day post‐treatment, compared to the control group, the lumen area and plaque area significantly increased by 272.3% and decreased by 51.8%, respectively. SDT mediated by PHPMR NPs not only reduced the density of neovessels by 59.1% but also decreased the percentage of intraplaque hemorrhage by 66.2%, thereby stabilizing the plaque and demonstrating high therapeutic biocompatibility.

## 5 CONCLUSION

In conclusion, recent development of various mitochondria‐targeting theranostic agents for different diseases, mainly cancer, have been reviewed and classified according to the involved imaging modalities and show good imaging ability and treatment effect, demonstrating that mitochondrion is a promising theranostic target. Among these agents, optical ones and related treatment approaches, including PTT and PDT, are attracting more attentions and research input for their unique advancement, such as non‐ionizing radiation, regional therapeutic effects, low toxicity for normal tissues, and low‐cost equipment. However, limited by in vivo penetration depth, possible clinical applications of optical theranostic strategies are restricted to superficial tissues and related disorders. Efforts to tackle such difficulties have been devoted, including employing fluorophores with longer fluorescence wavelengths in NIR‐IIb (1500–1700 nm) window and improving the sensitivities and image process algorithms to clarify the scattering optical signals. These advances could enlighten the design of optical mitochondria‐targeting theranostic agents.

Organic and inorganic heavy metal nanoparticles consist of a relatively large part of reviewed theranostic agents with excellent performance. However, long‐term safety concerns about these materials may be raised considering their slow in vivo metabolic rate and possible leakage of heavy metals, which could limit their clinical translation. Theranostic agents in small molecule form usually possess well defined structure and better biocompatibility, endowing them with higher potential in clinical application of mitochondria‐related disease treatment.

More modalities of imaging and therapy, such as PET, SPECT, radionuclide therapy, and others, may be considered in the development of future mitochondria‐targeting theranostic agent to provide more dimensions of information and treatment options on relevant disorders, enriching the arsenal for combating mitochondria‐related diseases. Radiopharmaceuticals could be another trending direction for mitochondria‐targeting theranostic development as radionuclide labeled mitochondria‐targeting probe has already been reported and showed outstanding targeted imaging ability. Molecular imaging is a powerful and efficient tool in drug screening and discovery,^[^ ^171^ ^]^ and once candidates with excellent imaging capability of mitochondria related diseases are obtained in this manner, therapeutic agents with similar targeting ability may be rapidly and readily derived by therapeutic radionuclide labeling (^131^I, ^177^Lu, ^225^Ac, etc.) to exert localized lesion treatment, limiting radiation within desired tissues.

## CONFLICT OF INTEREST STATEMENT

The authors declare no conflicts of interest.

## References

[1] R. McFarland , R. W. Taylor , D. M. Turnbull , Curr. Top. Dev. Biol. 2007, 77, 113. 17222702 10.1016/S0070-2153(06)77005-3

[2] B. G. Heerdt , N. A. Houston , L. H. Augenlicht , Cancer Res. 2006, 66, 1591. 16452217 10.1158/0008-5472.CAN-05-2717

[3] F. G. Tahrir , D. Langford , S. Amini , T. M. Ahooyi , K. Khalili , J. Cell. Physiol. 2019, 234, 8122. 30417391 10.1002/jcp.27597 PMC6395499

[4] a) J. Li , J. Lu , Y. Zhou , Biomed. Res. Int. 2017, 2017, 5246853; 28638829 10.1155/2017/5246853 PMC5468586

[5] S.‐Y. Jeong , D.‐W. Seol , BMB Rep. 2008, 41, 11. 18304445 10.5483/bmbrep.2008.41.1.011

[6] W. Y. Kim , M. Won , S. Koo , X. Zhang , J. S. Kim , Nano‐Micro Lett. 2021, 13, 168. 10.1007/s40820-021-00689-1 PMC8342730 34355274

[7] P. Rai , S. Mallidi , X. Zheng , R. Rahmanzadeh , Y. Mir , S. Elrington , A. Khurshid , T. Hasan , Adv. Drug Delivery Rev. 2010, 62, 1094. 10.1016/j.addr.2010.09.002 PMC2991599 20858520

[8] a) S. G. E. Andersson , A. Zomorodipour , J. O. Andersson , T. Sicheritz‐Ponten , U. C. M. Alsmark , R. M. Podowski , A. K. Naslund , A. S. Eriksson , H. H. Winkler , C. G. Kurland , Nature 1998, 396, 133; 9823893 10.1038/24094

[9] N. Lane , W. Martin , Nature 2010, 467, 929. 20962839 10.1038/nature09486

[10] a) P. R. Rich , A. Marechal , Essays Biochem. 2010, 47, 1; 20533897 10.1042/bse0470001

[11] X. Guo , N. Yang , W. Ji , H. Zhang , X. Dong , Z. Zhou , L. Li , H. M. Shen , S. Q. Yao , W. Huang , Adv. Mater. 2021, 33, e2007778. 34510563 10.1002/adma.202007778

[12] G. Bagkos , K. Koufopoulos , C. Piperi , Curr. Pharm. Des. 2014, 20, 4570. 24372303 10.2174/1381612819666131230124334

[13] a) S. E. Horvath , G. Daum , Prog. Lipid Res. 2013, 52, 590; 24007978 10.1016/j.plipres.2013.07.002

[14] I. Vercellino , L. A. Sazanov , Nat. Rev. Mol. Cell Biol. 2022, 23, 141. 34621061 10.1038/s41580-021-00415-0

[15] a) J. R. Friedman , J. Nunnari , Nature 2014, 505, 335; 24429632 10.1038/nature12985 PMC4075653

[16] Y. Yamada , H. Harashima , Adv. Drug Delivery Rev. 2008, 60, 1439. 10.1016/j.addr.2008.04.016 18655816

[17] H. B. Ngo , J. T. Kaiser , D. C. Chan , Nat. Struct. Mol. Biol. 2011, 18, 1290. 22037171 10.1038/nsmb.2159 PMC3210390

[18] D. E. Green , D. H. Maclennan , Bioscience 1969, 19, 213.

[19] P. Mishra , D. C. Chan , Nat. Rev. Mol. Cell Biol. 2014, 15, 634. 25237825 10.1038/nrm3877 PMC4250044

[20] P. Samangouei , G. E. Crespo‐Avilan , H. Cabrera‐Fuentes , S. Hernandez‐Resendiz , N. I. Ismail , K. B. Katwadi , W. A. Boisvert , D. J. Hausenloy , Cond. Med. 2018, 1, 239. 30338314 PMC6191188

[21] T. Koshiba , S. A. Detmer , J. T. Kaiser , H. C. Chen , J. M. McCaffery , D. C. Chan , Science 2004, 305, 858. 15297672 10.1126/science.1099793

[22] a) J. A. Stefely , N. W. Kwiecien , E. C. Freiberger , A. L. Richards , A. Jochem , M. J. P. Rush , A. Ulbrich , K. P. Robinson , P. D. Hutchins , M. T. Veling , X. Guo , Z. A. Kemmerer , K. J. Connors , E. A. Trujillo , J. Sokol , H. Marx , M. S. Westphall , A. S. Hebert , D. J. Pagliarini , J. J. Coon , Nat. Biotechnol. 2016, 34, 1191; 27669165 10.1038/nbt.3683 PMC5101133

[23] M. F. Lopez , B. S. Kristal , E. Chernokalskaya , A. Lazarev , A. I. Shestopalov , A. Bogdanova , M. Robinson , Electrophoresis 2000, 21, 3427. 11079563 10.1002/1522-2683(20001001)21:16<3427::AID-ELPS3427>3.0.CO;2-L

[24] a) C. W. Birky , Proc. Natl. Acad. Sci. U. S. A. 1995, 92, 11331; 8524780 10.1073/pnas.92.25.11331 PMC40394

[25] J. Nunnari , A. Suomalainen , Cell 2012, 148, 1145. 22424226 10.1016/j.cell.2012.02.035 PMC5381524

[26] a) S. V. Mangrulkar , N. L. Wankhede , M. B. Kale , A. B. Upaganlawar , B. G. Taksande , M. J. Umekar , M. K. Anwer , H. G. Dailah , S. Mohan , T. Behl , Neurotox. Res. 2023, 41, 708. 37162686 10.1007/s12640-023-00647-2

[27] L. Granat , R. J. Hunt , J. M. Bateman , Philos. Trans. R. Soc., B 2020, 375, 20190415. 10.1098/rstb.2019.0415 PMC7209953 32362256

[28] a) P. Onyango , I. Celic , J. M. McCaffery , J. D. Boeke , A. P. Feinberg , Proc. Natl. Acad. Sci. U. S. A. 2002, 99, 13653; 12374852 10.1073/pnas.222538099 PMC129731

[29] M. Morigi , L. Perico , C. Rota , L. Longaretti , S. Conti , D. Rottoli , R. Novelli , G. Remuzzi , A. Benigni , J. Clin. Invest. 2015, 125, 715. 25607838 10.1172/JCI77632 PMC4319434

[30] A. T. Turer , J. A. Hill , Am. J. Cardiol. 2010, 106, 360. 20643246 10.1016/j.amjcard.2010.03.032 PMC2957093

[31] J. Wang , H. Zhou , Acta Pharm. Sin. B 2020, 10, 1866. 33163341 10.1016/j.apsb.2020.03.004 PMC7606115

[32] T. Saito , J. Nah , S. I. Oka , R. Mukai , Y. Monden , Y. Maejima , Y. Ikeda , S. Sciarretta , T. Liu , H. Li , E. Baljinnyam , D. Fraidenraich , L. Fritzky , P. Zhai , S. Ichinose , M. Isobe , C. P. Hsu , M. Kundu , J. Sadoshima , J. Clin. Invest. 2019, 129, 802. 30511961 10.1172/JCI122035 PMC6355232

[33] T. Sun , W. Ding , T. Xu , X. Ao , T. Yu , M. Li , Y. Liu , X. Zhang , L. Hou , J. Wang , Antioxid. Redox Signal. 2019, 31, 1177. 31456416 10.1089/ars.2019.7734

[34] A. Fu , Y. Hou , Z. Yu , Z. Zhao , Z. Liu , Int. J. Biol. Sci. 2019, 15, 2707. 31754341 10.7150/ijbs.38104 PMC6854369

[35] I. A. Barbosa , N. G. Machado , A. J. Skildum , P. M. Scott , P. J. Oliveira , Biochim. Biophys. Acta, Rev. Cancer 2012, 1826, 238. 10.1016/j.bbcan.2012.04.005 22554970

[36] L. D. Zorova , V. A. Popkov , E. Y. Plotnikov , D. N. Silachev , I. B. Pevzner , S. S. Jankauskas , V. A. Babenko , S. D. Zorov , A. V. Balakireva , M. Juhaszova , S. J. Sollott , D. B. Zorov , Anal. Biochem. 2018, 552, 50. 28711444 10.1016/j.ab.2017.07.009 PMC5792320

[37] a) A. A. Gerencser , C. Chinopoulos , M. J. Birket , M. Jastroch , C. Vitelli , D. G. Nicholls , M. D. Brand , J. Physiol. 2012, 590, 2845; 22495585 10.1113/jphysiol.2012.228387 PMC3448152

[38] S. S. Liew , X. Qin , J. Zhou , L. Li , W. Huang , S. Q. Yao , Angew. Chem., Int. Ed. 2021, 60, 2232. 10.1002/anie.201915826 32128948

[39] C. Gianpiero , D. Anis , R. Aikaterini , T. Eirini , V. S. Ioannis , F. G. Dimitrios , T. John , MedChemComm 2017, 8, 67. 30108691 10.1039/c6md00383d PMC6072302

[40] Q. Q. Li , Y. Hamamoto , G. Kwek , B. G. Xing , Y. X. Li , S. Ito , Angew. Chem., Int. Ed. 2022, 61, e202112638. 10.1002/anie.202112638 34863045

[41] H. Tanaka , M. Yamamoto , N. Hashimoto , M. Miyakoshi , S. Tamakawa , M. Yoshie , Y. Tokusashi , K. Yokoyama , Y. Yaginuma , K. Ogawa , Cancer Res. 2006, 66, 11263. 17145871 10.1158/0008-5472.CAN-06-1699

[42] a) N. C. Denko , Nat. Rev. Cancer 2008, 8, 705; 19143055 10.1038/nrc2468

[43] G. L. Semenza , IUBMB Life 2008, 60, 591. 18506846 10.1002/iub.93

[44] R. Iurlaro , C. Lucia Leon‐Annicchiarico , C. Munoz‐Pinedo , Methods Enzymol. 2014, 542, 59. 24862260 10.1016/B978-0-12-416618-9.00003-0

[45] F. Morrish , D. Hockenbery , Perspect. Med. 2014, 4, a014225. 10.1101/cshperspect.a014225 PMC3996374 24789872

[46] D. R. Wonsey , K. I. Zeller , C. V. Dang , Proc. Natl. Acad. Sci. U. S. A. 2002, 99, 6649. 12011429 10.1073/pnas.102523299 PMC124457

[47] F. Li , Y. Y. Wang , K. I. Zeller , J. J. Potter , D. R. Wonsey , K. A. O'Donnell , J. W. Kim , J. T. Yustein , L. A. Lee , C. V. Dang , Mol. Cell. Biol. 2005, 25, 6225. 15988031 10.1128/MCB.25.14.6225-6234.2005 PMC1168798

[48] J. A. Kashatus , A. Nascimento , L. J. Myers , A. Sher , F. L. Byrne , K. L. Hoehn , C. M. Counter , D. F. Kashatus , Mol. Cell 2015, 57, 537. 25658205 10.1016/j.molcel.2015.01.002 PMC4393013

[49] S. Ohsawa , Y. Sato , M. Enomoto , M. Nakamura , A. Betsumiya , T. Igaki , Nature 2012, 490, 547. 23023132 10.1038/nature11452

[50] C. Evangelisti , D. de Biase , I. Kurelac , C. Ceccarelli , H. Prokisch , T. Meitinger , P. Caria , R. Vanni , G. Romeo , G. Tallini , G. Gasparre , E. Bonora , BMC Cancer 2015, 15, 157. 25880213 10.1186/s12885-015-1122-3 PMC4374372

[51] K. Kawauchi , K. Araki , K. Tobiume , N. Tanaka , Nat. Cell Biol. 2008, 10, 611. 18391940 10.1038/ncb1724

[52] J.‐Q. Chen , J. Russo , Biochim. Biophys. Acta, Rev. Cancer 2012, 1826, 370. 10.1016/j.bbcan.2012.06.004 22750268

[53] H. Cho , Y. Y. Cho , M. S. Shim , J. Y. Lee , H. S. Lee , H. C. Kang , Biochim. Biophys. Acta, Mol. Basis Dis. 2020, 1866, 165808. 32333953 10.1016/j.bbadis.2020.165808

[54] R. O. Poyton , K. A. Ball , P. R. Castello , Trends Endocrinol. Metab. 2009, 20, 332. 19733481 10.1016/j.tem.2009.04.001

[55] a) T. Ismail , Y. Kim , H. Lee , D.‐S. Lee , H.‐S. Lee , Int. J. Mol. Sci. 2019, 20, 4407; 31500275 10.3390/ijms20184407 PMC6770548

[56] Y. Chen , H. Zhang , H. J. Zhou , W. Ji , W. Min , Cancers 2016, 8, 40. 27023612 10.3390/cancers8040040 PMC4846849

[57] M. Abate , A. Festa , M. Falco , A. Lombardi , A. Luce , A. Grimaldi , S. Zappavigna , P. Sperlongano , C. Irace , M. Caraglia , G. Misso , Semin. Cell Dev. Biol. 2020, 98, 139. 31154010 10.1016/j.semcdb.2019.05.022

[58] a) B. Vurusaner , G. Poli , H. Basaga , Biol. Med. 2012, 52, 7; 10.1016/j.freeradbiomed.2011.09.035 22019631

[59] S. Lin , Y. Li , A. A. Zamyatnin , J. Werner , A. V. Bazhin , J. Cell. Physiol. 2018, 233, 5119. 29215746 10.1002/jcp.26356

[60] a) J. Wang , H. Zhang , J. Lv , Y. Zheng , M. Li , G. Yang , X. Wei , N. Li , H. Huang , T. Li , X. Qin , S. Li , C. Wu , W. Zhang , Y. Liu , H. Yang , Acta Biomater. 2023, 164, 522; 37072069 10.1016/j.actbio.2023.04.014

[61] S. K. Rout , V. Priya , A. Setia , A. K. Mehata , S. Mohan , M. Albratty , A. Najmi , A. M. Meraya , H. A. Makeen , M. M. Tambuwala , M. S. Muthu , Biomed. Pharmacother. 2022, 153, 113451. 36076564 10.1016/j.biopha.2022.113451

[62] a) M. Millard , D. Pathania , Y. Shabaik , L. Taheri , J. Deng , N. Neamati , PLoS One 2010, 5, e13131; 20957228 10.1371/journal.pone.0013131 PMC2949386

[63] G. Battogtokh , Y. S. Choi , D. S. Kang , S. J. Park , M. S. Shim , K. M. Huh , Y.‐Y. Cho , J. Y. Lee , H. S. Lee , H. C. Kang , Acta Pharm. Sin. B 2018, 8, 862. 30505656 10.1016/j.apsb.2018.05.006 PMC6251809

[64] Y. H. Sun , H. Zhang , G. Z. Lu , H. Wang , Y. Lu , L. Fan , Chin. Chem. Lett. 2023, 34, 107817.

[65] a) E. Kawamura , Y. Yamada , H. Harashima , Mitochondrion 2013, 13, 610; 24012978 10.1016/j.mito.2013.08.010

[66] H. Cheng , R.‐R. Zheng , G.‐L. Fan , J.‐H. Fan , L.‐P. Zhao , X.‐Y. Jiang , B. Yang , X.‐Y. Yu , S.‐Y. Li , X.‐Z. Zhang , Biomaterials 2019, 188, 1. 30312907 10.1016/j.biomaterials.2018.10.005

[67] Q. Li , J. Yang , C. Chen , X. Lin , M. Zhou , Z. Zhou , Y. Huang , J. Controlled Release 2020, 325, 38. 10.1016/j.jconrel.2020.06.010 32598957

[68] a) Y. Wang , A. G. Cheetham , G. Angacian , H. Su , L. S. Xie , H. G. Cui , Adv. Drug Delivery Rev. 2017, 110‐111, 112; 10.1016/j.addr.2016.06.015 PMC5199637 27370248

[69] R. Lin , P. C. Zhang , A. G. Cheetham , J. Walston , P. Abadir , H. G. Cui , Bioconjug. Chem. 2015, 26, 71. 25547808 10.1021/bc500408p PMC4306504

[70] G. Z. Zhu , X. Y. Chen , Adv. Drug Delivery Rev. 2018, 134, 65. 10.1016/j.addr.2018.08.005 PMC6239901 30125604

[71] a) J. Lebeau , T. K. Rainbolt , R. L. Wiseman , Int. Rev. Cell Mol. Biol. 2018, 340, 79; 30072094 10.1016/bs.ircmb.2018.05.003 PMC6402875

[72] J. Qi , J. Deng , K. Qian , L. Tian , J. Li , K. He , X. Huang , Z. Cheng , Y. Zheng , Y. Wang , Eur. J. Med. Chem. 2017, 134, 34. 28395152 10.1016/j.ejmech.2017.04.009

[73] a) Y. Li , J. Zhuang , Y. Lu , N. Li , M. Gu , J. Xia , N. Zhao , B. Z. Tang , ACS Nano 2021, 15, 20453; 34843216 10.1021/acsnano.1c08928

[74] A. Yuan , J. Wu , X. Tang , L. Zhao , F. Xu , Y. Hu , J. Pharm. Sci. 2013, 102, 6. 23132644 10.1002/jps.23356

[75] Q. Wan , R. Zhang , Z. Zhuang , Y. Li , Y. Huang , Z. Wang , W. Zhang , J. Hou , B. Z. Tang , Adv. Funct. Mater. 2020, 30, 2002057.

[76] a) T. J. Dougherty , C. J. Gomer , B. W. Henderson , G. Jori , D. Kessel , M. Korbelik , J. Moan , Q. Peng , J. Natl. Cancer Inst. 1998, 90, 889; 9637138 10.1093/jnci/90.12.889 PMC4592754

[77] X. Deng , Z. Shao , Y. Zhao , Adv. Sci. 2021, 8, 2002504. 10.1002/advs.202002504 PMC7856884 33552860

[78] J. T. Robinson , S. M. Tabakman , Y. Liang , H. Wang , H. S. Casalongue , V. Daniel , H. Dai , J. Am. Chem. Soc. 2011, 133, 6825. 21476500 10.1021/ja2010175

[79] W. Guo , C. Guo , N. Zheng , T. Sun , S. Liu , Adv. Mater. 2017, 29, 1604157. 10.1002/adma.201604157 27874227

[80] C. Ruan , C. Liu , H. Hu , X.‐L. Guo , B.‐P. Jiang , H. Liang , X.‐C. Shen , Chem. Sci. 2019, 10, 4699. 31123581 10.1039/c9sc00375d PMC6496981

[81] T. J. Wigmore , K. Mohammed , S. Jhanji , Anesthesiology 2016, 124, 69. 26556730 10.1097/ALN.0000000000000936

[82] a) W. Hartwig , J. Werner , D. Jaeger , J. Debus , M. W. Buechler , Lancet Oncol. 2013, 14, e476; 24079875 10.1016/S1470-2045(13)70172-4

[83] H. Tsujimoto , Y. Yaguchi , I. Kumano , R. Takahata , S. Ono , K. Hase , World J. Surg. 2012, 36, 327. 22187132 10.1007/s00268-011-1387-x

[84] a) J. Wang , X. L. Wang , K. Yang , S. J. Hu , W. H. Wang , Biosensors 2022, 12, 683;36140068

[85] a) P. S. Low , S. Singhal , M. Srinivasarao , Curr. Opin. Chem. Biol. 2018, 45, 64; 29579618 10.1016/j.cbpa.2018.03.002

[86] H. C. Suen , M. K. Y. Hsin , J. Thorac. Cardiovasc. Surg. 2022, 164, 581. 33618874 10.1016/j.jtcvs.2020.09.025

[87] S. Tohme , R. L. Simmons , A. Tsung , Cancer Res. 2017, 77, 1548. 28330928 10.1158/0008-5472.CAN-16-1536 PMC5380551

[88] a) M. Peng , Y. Ying , Z. Zhang , L. Liu , W. Wang , Cancers 2023, 15, 2448; 37173915 10.3390/cancers15092448 PMC10177210

[89] a) D.‐Y. Oh , K.‐H. Lee , D.‐W. Lee , J. Yoon , T.‐Y. Kim , J.‐H. Bang , A.‐R. Nam , K.‐S. Oh , J.‐M. Kim , Y. Lee , V. Guthrie , P. McCoon , W. Li , S. Wu , Q. Zhang , M. C. Rebelatto , J. W. Kim , Lancet Gastroenterol. Hepatol. 2022, 7, 522;35278356

[90] A. R. Cantelmo , L.‐C. Conradi , A. Brajic , J. Goveia , J. Kalucka , A. Pircher , P. Chaturvedi , J. Hol , B. Thienpont , L.‐A. Teuwen , S. Schoors , B. Boeckx , J. Vriens , A. Kuchnio , K. Veys , B. Cruys , L. Finotto , L. Treps , T. E. Stav‐Noraas , F. Bifari , P. Stapor , I. Decimo , K. Kampen , K. De Bock , G. Haraldsen , L. Schoonjans , T. Rabelink , G. Eelen , B. Ghesquiere , J. Rehman , et al., Cancer Cell 2016, 30, 968. 27866851 10.1016/j.ccell.2016.10.006 PMC5675554

[91] Y. Zhang , J. Gao , X. Wang , S. Deng , H. Ye , W. Guan , M. Wu , S. Zhu , Y. Yu , W. Han , Cancer Biol. Ther. 2015, 16, 1775. 26479470 10.1080/15384047.2015.1095404 PMC4847813

[92] a) L. Amable , Pharmacol. Res. 2016, 106, 27; 26804248 10.1016/j.phrs.2016.01.001

[93] a) F. Yu , Y. Tu , S. Luo , X. Xiao , W. Yao , M. Jiang , X. Jiang , R. Yang , Y. Yuan , Nano Lett. 2021, 21, 2216; 33635657 10.1021/acs.nanolett.0c05028

[94] a) G. Song , L. Cheng , Y. Chao , K. Yang , Z. Liu , Adv. Mater. 2017, 29, 1700996, 1700996; 10.1002/adma.201700996 28643452

[95] E. J. Moding , M. B. Kastan , D. G. Kirsch , Nat. Rev. Drug Discovery 2013, 12, 526. 23812271 10.1038/nrd4003 PMC3906736

[96] H. E. Barker , J. T. E. Paget , A. A. Khan , K. J. Harrington , Nat. Rev. Cancer 2015, 15, 409. 26105538 10.1038/nrc3958 PMC4896389

[97] a) Q. Chen , J. Chen , Z. Yang , J. Xu , L. Xu , C. Liang , X. Han , Z. Liu , Adv. Mater. 2019, 31, 1802228; 10.1002/adma.201802228 30663118

[98] a) A. Hamacher‐Brady , H. A. Stein , S. Turschner , I. Toegel , R. Mora , N. Jennewein , T. Efferth , R. Eils , N. R. Brady , J. Biol. Chem. 2011, 286, 6587; 21149439 10.1074/jbc.M110.210047 PMC3057810

[99] Z. Tang , Y. Liu , M. He , W. Bu , Angew. Chem., Int. Ed. 2019, 58, 946. 10.1002/anie.201805664 30048028

[100] a) W. Zhang , X. Hu , Q. Shen , D. Xing , Nat. Commun. 2019, 10, 2597; 31182721 10.1038/s41467-019-10186-0 PMC6557808

[101] J. An , Y.‐G. Hu , C. Li , X.‐L. Hou , K. Cheng , B. Zhang , R.‐Y. Zhang , D.‐Y. Li , S.‐J. Liu , B. Liu , D. Zhu , Y.‐D. Zhao , Biomaterials 2020, 230, 119636. 31785776 10.1016/j.biomaterials.2019.119636

[102] M. Xu , L. Zhou , L. Zheng , Q. Zhou , K. Liu , Y. Mao , S. Song , Cancer Lett. 2021, 497, 229. 33122099 10.1016/j.canlet.2020.10.037

[103] a) X. Qian , Y. Zheng , Y. Chen , Adv. Mater. 2016, 28, 8097; 27384408 10.1002/adma.201602012

[104] S. Liang , X. Deng , G. Xu , X. Xiao , M. Wang , X. Guo , P. a. Ma , Z. Cheng , D. Zhang , J. Lin , Adv. Funct. Mater. 2020, 30, 1908598.

[105] Q. Y. Zhang , Q. H. Luo , Z. M. Liu , M. C. Sun , X. Dong , Chem. Eng. J. 2023, 457, 141225.

[106] X. Y. Deng , Z. W. Shao , Y. L. Zhao , Adv. Sci. 2021, 8, 2002504. 10.1002/advs.202002504 PMC7856884 33552860

[107] a) T. W. Li , L. Liu , P. P. Xu , P. Yuan , Y. L. Tian , Q. Cheng , L. F. Yan , Adv. Ther. 2021, 4, 2000240.

[108] G. Feng , G. Q. Zhang , D. Ding , Chem. Soc. Rev. 2020, 49, 8179. 33196726 10.1039/d0cs00671h

[109] A. Kohler , J. S. Wilson , R. H. Friend , Adv. Mater. 2002, 14, 701.

[110] F. Hu , L. Shi , W. Min , Nat. Methods 2019, 16, 830. 31471618 10.1038/s41592-019-0538-0

[111] G. Hong , A. L. Antaris , H. Dai , Nat. Biomed. Eng. 2017, 1, 0010.

[112] a) T. F. Massoud , S. S. Gambhir , Genes Dev. 2003, 17, 545; 12629038 10.1101/gad.1047403

[113] a) Y. Chen , S. Wang , F. Zhang , Nat. Rev. Bioeng. 2023, 1, 60;

[114] S. He , J. Song , J. Qu , Z. Cheng , Chem. Soc. Rev. 2018, 47, 4258. 29725670 10.1039/c8cs00234g

[115] A. L. Antaris , H. Chen , K. Cheng , Y. Sun , G. Hong , C. Qu , S. Diao , Z. Deng , X. Hu , B. Zhang , X. Zhang , O. K. Yaghi , Z. R. Alamparambil , X. Hong , Z. Cheng , H. Dai , Nat. Mater. 2016, 15, 235. 26595119 10.1038/nmat4476

[116] K. Cheng , H. Chen , C. H. Jenkins , G. Zhang , W. Zhao , Z. Zhang , F. Han , J. Fung , M. Yang , Y. Jiang , L. Xing , Z. Cheng , ACS Nano 2017, 11, 12276. 29202225 10.1021/acsnano.7b05966

[117] V. R. Fantin , M. J. Berardi , L. Scorrano , S. J. Korsmeyer , P. Leder , Cancer Cell 2002, 2, 29. 12150823 10.1016/s1535-6108(02)00082-x

[118] L. Zheng , Z. Wang , X. Zhang , Y. Zhou , A. Ji , H. Lou , X. Liu , H. Chen , Z. Cheng , J. Med. Chem. 2022, 65, 497. 34937337 10.1021/acs.jmedchem.1c01660

[119] H. Chen , J. Wang , X. Feng , M. Zhu , S. Hoffmann , A. Hsu , K. Qian , D. Huang , F. Zhao , W. Liu , H. Zhang , Z. Cheng , Chem. Sci. 2019, 10, 7946. 31853349 10.1039/c9sc01410a PMC6836573

[120] K. Qian , H. Chen , C. Qu , J. Qi , B. Du , T. Ko , Z. Xiang , M. Kandawa‐Schulz , Y. Wang , Z. Cheng , Nanomed. Nanotechnol. Biol. Med. 2020, 23, 102087. 10.1016/j.nano.2019.102087 31454551

[121] B. Guo , M. Wu , Q. Shi , T. Dai , S. Xu , J. Jiang , B. Liu , Chem. Mater. 2020, 32, 4681.

[122] L. Zhang , F. L. Jiang , Y. Liu , P. Jiang , J. Phys. Chem. Lett. 2022, 13, 3462. 35413203 10.1021/acs.jpclett.2c00541

[123] N. Mizushima , T. Yoshimori , B. Levine , Cell 2010, 140, 313. 20144757 10.1016/j.cell.2010.01.028 PMC2852113

[124] D. Ma , Q. Zong , Y. Du , F. Yu , X. Xiao , R. Sun , Y. Guo , X. Wei , Y. Yuan , Acta Biomater. 2021, 135, 628. 34371167 10.1016/j.actbio.2021.08.002

[125] Z. Liu , H. Wang , C. Sun , Y. He , T. Xia , J. Wang , X. Xiong , Q. Zhang , S. Yang , L. Liu , Front. Pharmacol. 2022, 13, 829684. 35281928 10.3389/fphar.2022.829684 PMC8905922

[126] H. Heng , G. Song , X. Cai , J. Sun , K. Du , X. Zhang , X. Wang , F. Feng , S. Wang , Angew. Chem., Int. Ed. 2022, 61, e202203444. 10.1002/anie.202203444 35763340

[127] M. Yao , Y. Lu , L. Shi , Y. Huang , Q. Zhang , J. Tan , P. Hu , J. Zhang , G. Luo , N. Zhang , Bioact. Mater. 2022, 9, 168. 34820564 10.1016/j.bioactmat.2021.07.011 PMC8586025

[128] Y. Zheng , Q. Li , J. Wu , Z. Luo , W. Zhou , A. Li , Y. Chen , T. Rouzi , T. Tian , H. Zhou , X. Zeng , Y. Li , X. Cheng , Y. Wei , Z. Deng , F. Zhou , X. Hong , Chem. Sci. 2020, 12, 1843. 34163948 10.1039/d0sc04727a PMC8179124

[129] H. Zhou , X. Zeng , A. Li , W. Zhou , L. Tang , W. Hu , Q. Fan , X. Meng , H. Deng , L. Duan , Y. Li , Z. Deng , X. Hong , Y. Xiao , Nat. Commun. 2020, 11, 6183. 33273452 10.1038/s41467-020-19945-w PMC7713230

[130] a) J. Zhou , Q. Liu , W. Feng , Y. Sun , F. Li , Chem. Rev. 2015, 115, 395; 25492128 10.1021/cr400478f

[131] Q. Wang , J. Xu , R. Geng , J. Cai , J. Li , C. Xie , W. Tang , Q. Shen , W. Huang , Q. Fan , Biomaterials 2020, 231, 119671. 31855624 10.1016/j.biomaterials.2019.119671

[132] L. K. McKenzie , I. V. Sazanovich , E. Baggaley , M. Bonneau , V. Guerchais , J. A. Williams , J. A. Weinstein , H. E. Bryant , Chemistry 2017, 23, 234. 27740703 10.1002/chem.201604792 PMC5248616

[133] a) Y. You , S. Lee , T. Kim , K. Ohkubo , W. S. Chae , S. Fukuzumi , G. J. Jhon , W. Nam , S. J. Lippard , J. Am. Chem. Soc. 2011, 133, 18328; 22023085 10.1021/ja207163r PMC3212631

[134] S. Yi , Z. Lu , J. Zhang , J. Wang , Z. Xie , L. Hou , ACS Appl. Mater. Interfaces 2019, 11, 15276. 30968687 10.1021/acsami.9b01205

[135] J. Liu , A. W. Prentice , G. J. Clarkson , J. M. Woolley , V. G. Stavros , M. J. Paterson , P. J. Sadler , Adv. Mater. 2023, 35, e2210363. 36787500 10.1002/adma.202210363

[136] J. Liu , G. Ding , S. Chen , C. Xue , M. Chen , X. Wu , Q. Yuan , J. Zheng , R. Yang , ACS Appl. Mater. Interfaces 2021, 13, 9681. 33606499 10.1021/acsami.0c21681

[137] Z. Zheng , X. Chen , Y. Ma , R. Dai , S. Wu , T. Wang , J. Xing , J. Gao , R. Zhang , Small 2022, 18, e2203531. 35962758 10.1002/smll.202203531

[138] N. C. Burton , M. Patel , S. Morscher , W. H. P. Driessen , J. Claussen , N. Beziere , T. Jetzfellner , A. Taruttis , D. Razansky , B. Bednar , V. Ntziachristos , Neuroimage 2013, 65, 522. 23026761 10.1016/j.neuroimage.2012.09.053

[139] a) M. H. Xu , L. H. V. Wang , Rev. Sci. Instrum. 2006, 77, 041101;

[140] C. Kim , D. Razansky , IEEE Pulse 2015, 6, 3. 10.1109/MPUL.2015.2409103 25974916

[141] L. V. Wang , S. Hu , Science 2012, 335, 1458. 22442475 10.1126/science.1216210 PMC3322413

[142] B. Park , D. Oh , J. Kim , C. Kim , Nano Converg. 2023, 10, 29. 37335405 10.1186/s40580-023-00377-3 PMC10279631

[143] C. Ji , W. Cheng , Y. Hu , Y. Liu , F. Liu , M. Yin , Nano Today 2021, 36, 101020.

[144] a) H. Kabasawa , Magn. Reson. Med. Sci. 2022, 21, 71; 33867419 10.2463/mrms.rev.2021-0011 PMC9199974

[145] A. P. Koretsky , Ann. N. Y. Acad. Sci. 2002, 961, 203. 12081900 10.1111/j.1749-6632.2002.tb03084.x

[146] a) W. Huang , L. A. Tudorica , X. Li , S. B. Thakur , Y. Chen , E. A. Morris , I. J. Tagge , M. E. Korenblit , W. D. Rooney , J. A. Koutcher , C. S. j r. Springer , Radiology 2011, 261, 394; 21828189 10.1148/radiol.11102413 PMC3198224

[147] C. Kuhl , Radiology 2007, 244, 356. 17641361 10.1148/radiol.2442051620

[148] D. Patra , S. Kumar , P. Kumar , I. Chakraborty , B. Basheer , R. Shunmugam , Biomacromolecules 2022, 23, 3198. 35767830 10.1021/acs.biomac.2c00302

[149] A. Boss , S. Bisdas , A. Kolb , M. Hofmann , U. Ernemann , C. D. Claussen , C. Pfannenberg , B. J. Pichler , M. Reimold , L. Stegger , J. Nucl. Med. 2010, 51, 1198. 20660388 10.2967/jnumed.110.074773

[150] Y. Liu , G. Yu , M. Tian , H. Zhang , Contrast Media Mol. Imaging 2011, 6, 169. 21246711 10.1002/cmmi.428

[151] a) K. Wang , C. W. Chi , Z. H. Hu , M. H. Liu , H. Hui , W. T. Shang , D. Peng , S. Zhang , J. Z. Ye , H. X. Liu , J. Tian , Engineering 2015, 1, 309;

[152] Y. M. Yang , J. Mu , B. G. Xing , Wiley Interdiscip. Rev.: Nanomed. Nanobiotechnol. 2017, 9, e1408. 10.1002/wnan.1408 27094696

[153] T. Zhang , J. Zhang , F. B. Wang , H. Cao , D. Zhu , X. Chen , C. Xu , X. Yang , W. Huang , Z. Wang , J. Wang , Z. He , Z. Zheng , J. W. Y. Lam , B. Z. Tang , Adv. Funct. Mater. 2022, 32, 2110526.

[154] N. Song , Z. Zhang , P. Liu , D. Dai , C. Chen , Y. Li , L. Wang , T. Han , Y. W. Yang , D. Wang , B. Z. Tang , Adv. Funct. Mater. 2021, 31, 2009924.

[155] J. Shin , Y. Xu , S. Koo , J. H. Lim , J. Y. Lee , A. Sharma , Y. Sun , J. S. Kim , Matter 2021, 4, 2508.

[156] a) K. Cai , W. Zhang , M. F. Foda , X. Li , J. Zhang , Y. Zhong , H. Liang , H. Li , H. Han , T. Zhai , Small 2020, 16, e2002748; 32780938 10.1002/smll.202002748

[157] J. Zhang , X. Yin , C. Li , X. Yin , Q. Xue , L. Ding , J. Ju , J. Ma , Y. Zhu , D. Du , R. L. Reis , Y. Wang , Adv. Mater. 2022, 34, e2110690. 35275432 10.1002/adma.202110690

[158] E. M. McNeil , D. W. Melton , Nucleic Acids Res. 2012, 40, 9990. 22941649 10.1093/nar/gks818 PMC3488251

[159] W. Zhang , X. F. Du , B. Liu , C. Li , J. Long , M. X. Zhao , Z. Yao , X. J. Liang , Y. Lai , ACS Nano 2022, 16, 1421. 34962119 10.1021/acsnano.1c09555

[160] T. Matsunaga , D. Mu , C. H. Park , J. T. Reardon , A. Sancar , J. Biol. Chem. 1995, 270, 20862. 7657672 10.1074/jbc.270.35.20862

[161] Y. Wen , J. Hu , J. Liu , M. Li , Chem. Mater. 2021, 33, 7089.

[162] W. S. Seo , J. H. Lee , X. Sun , Y. Suzuki , D. Mann , Z. Liu , M. Terashima , P. C. Yang , M. V. McConnell , D. G. Nishimura , H. Dai , Nat. Mater. 2006, 5, 971. 17115025 10.1038/nmat1775

[163] a) A. J. Mieszawska , W. J. Mulder , Z. A. Fayad , D. P. Cormode , Mol. Pharm. 2013, 10, 831; 23360440 10.1021/mp3005885 PMC3593826

[164] J. Sun , J. Wang , W. Hu , Y. Wang , T. Chou , Q. Zhang , B. Zhang , Z. Yu , Y. Yang , L. Ren , H. Wang , ACS Appl. Mater. Interfaces 2021, 13, 10778. 33646767 10.1021/acsami.1c01238

[165] Q. Wang , G. Sui , X. Wu , D. Teng , L. Zhu , S. Guan , H. Ran , Z. Wang , H. Wang , Acta Biomater. 2020, 102, 367. 31778831 10.1016/j.actbio.2019.11.043

[166] N. S. Vasudev , A. R. Reynolds , Angiogenesis 2014, 17, 471. 24482243 10.1007/s10456-014-9420-y PMC4061466

[167] Q. Sun , Z. Zhou , N. Qiu , Y. Shen , Adv. Mater. 2017, 29, 1606628. 10.1002/adma.201606628 28234430

[168] a) S. Wang , P. Huang , X. Chen , Adv. Mater. 2016, 28, 7340; 27255214 10.1002/adma.201601498 PMC5014563

[169] B. Liang , B. Qiao , K. Yu , J. Cao , H. Zhou , Q. Jiang , Y. Zhong , Y. Cao , Z. Wang , Y. Zheng , ACS Appl. Mater. Interfaces 2022, 14, 13038. 35266691 10.1021/acsami.1c24708

[170] J. Yao , Z. Yang , L. Huang , C. Yang , J. Wang , Y. Cao , L. Hao , L. Zhang , J. Zhang , P. Li , Z. Wang , Y. Sun , H. Ran , Adv. Sci. 2021, 8, 2100850. 10.1002/advs.202100850 PMC8498883 34382370

[171] J. K. Willmann , N. van Bruggen , L. M. Dinkelborg , S. S. Gambhir , Nat. Rev. Drug Discovery 2008, 7, 591. 18591980 10.1038/nrd2290

